# Long Non-Coding RNA Expression Profiles in Hereditary Haemorrhagic Telangiectasia

**DOI:** 10.1371/journal.pone.0090272

**Published:** 2014-03-06

**Authors:** Pernille M. Tørring, Martin Jakob Larsen, Anette D. Kjeldsen, Lilian Bomme Ousager, Qihua Tan, Klaus Brusgaard

**Affiliations:** 1 Department of Clinical Genetics, Odense University Hospital, Odense, Denmark; 2 Department of Otorhinolaryngology, Odense University Hospital, Odense, Denmark; 3 Otorhinolaryngology, Institute of Clinical Research, University of Southern Denmark, Odense, Denmark; 4 Human Genetics, Institute of Clinical Research, University of Southern Denmark, Odense, Denmark; 5 Epidemiology, Biostatistics and Bio-demography, Institute of Public Health, University of Southern Denmark, Odense, Denmark; Harbin Institute of Technology, China

## Abstract

Hereditary Haemorrhagic Telangiectasia (HHT) is an autosomal dominantly inherited vascular disease characterized by the presence of mucocutaneous telangiectasia and arteriovenous malformations in visceral organs. HHT is predominantly caused by mutations in *ENG* and *ACVRL1*, which both belong to the TGF-β signalling pathway. The exact mechanism of how haploinsufficiency of *ENG* and *ACVRL1* leads to HHT manifestations remains to be identified. As long non-coding RNAs (lncRNAs) are increasingly recognized as key regulators of gene expression and constitute a sizable fraction of the human transcriptome, we wanted to assess whether lncRNAs play a role in the molecular pathogenesis of HHT manifestations. By microarray technology, we profiled lncRNA transcripts from HHT nasal telangiectasial and non-telangiectasial tissue using a paired design. The microarray probes were annotated using the GENCODE v.16 dataset, identifying 4,810 probes mapping to 2,811 lncRNAs. Comparing HHT telangiectasial tissue with HHT non-telangiectasial tissue, we identified 42 lncRNAs that are differentially expressed (q<0.001). Using GREAT, a tool that assumes cis-regulation, we showed that differently expressed lncRNAs are enriched for genomic loci involved in key pathways concerning HHT. Our study identified lncRNAs that are aberrantly expressed in HHT telangiectasia and indicates that lncRNAs may contribute to regulate protein-coding loci in HHT. These results suggest that the lncRNA component of the transcriptome deserves more attention in HHT. A deeper understanding of lncRNAs and their role in telangiectasia formation possesses potential for discovering therapeutic targets in HHT.

## Introduction

Hereditary Haemorrhagic Telangiectasia (HHT) is an autosomal dominantly inherited vascular disease characterized by the presence of mucocutaneous telangiectasia and arteriovenous malformations (AVMs) in visceral organs, primarily the lungs, liver and brain. The most common clinical manifestation is spontaneous and recurrent epistaxis [1], [2], originating from nasal telangiectasias, which can be difficult to prevent and can lead to severe anaemia. Moreover, pulmonary arteriovenous malformations (PAVMs) are seen in approximately one third of patients and are potentially lethal, due to haemorrhage or shunting of blood through these abnormal blood vessels, which can cause paradoxical embolisms and cerebral abscesses [2], [3]. The clinical manifestations of HHT are extremely variable, even between family members, and age-dependent penetrance is present. The reported prevalence in Denmark is around 1 in 6,500 [4]. Around 85% of clinically diagnosed HHT patients [5]–[7] carry a mutation in either *ENG* (HHT1) or *ACVRL1* (HHT2). Patients with mutations in *ENG* or *ACVRL1* are clinically similar, as all reported manifestations are known to occur in both. However, later onset and lower penetrance, less cerebral and pulmonary AVMs, but more liver involvement and a risk of developing pulmonary arterial hypertension, are observed in patients with *ACVRL1* mutations. Currently there is no cure for the disease; only symptomatic treatment is possible.

HHT manifestations are thought to result from an imbalance in the process of angiogenesis. Angiogenesis is the development of new capillary blood vessels from pre-existing vessels and is controlled by different cytokines such as vascular endothelial growth factor (VEGF) and transforming growth factor beta 1 (TGFβ1). *ENG* and *ACVRL1* encode receptor proteins: Endoglin and ALK1 (Activin A receptor type II-like 1) (SKR3) respectively, which are components of the TGF-β signalling pathway. The TGF-β signalling pathway plays a complex and important role in development and homeostasis of many organs, including the vascular system. In the latter, it is involved in cell proliferation, migration, extracellular matrix formation, vascular smooth muscle cell differentiation and vascular tone. As Endoglin and ALK1 proteins are predominantly expressed in endothelial cells, these are primary cellular targets of the disease. Thus, HHT manifestations are caused by a disturbance in the TGF-β signalling pathway [8]–[10]. The exact mechanism of how haploinsufficiency of *ENG* and *ACVRL1* leads to HHT manifestations remains to be identified.

Our knowledge of the non protein-coding part of the human transcriptome is expanding rapidly, and recently attention has shifted towards the most numerous but still poorly understood group—the long non-coding RNAs (lncRNAs). Long non-coding RNAs (lncRNAs) are defined as eukaryote RNAs longer than 200 nucleotides in length, without protein coding capacity. This arbitrary limit of 200 nucleotides distinguishes lncRNAs from small regulatory RNAs, such as microRNA and short interfering RNA (siRNA). Studies indicate that lncRNAs are generated through pathways similar to that of protein-coding genes, with similar histone-modification profiles, splicing signals, and exon/intron lengths [11]. LncRNAs have been shown to be involved in major mechanisms of transcriptional and post-transcriptional regulation, such as targeting transcription factors, initiating chromatin remodelling, directing methylation complexes and blocking nearby transcription [12]. LncRNAs appear to control expression of protein-coding genes through both *cis*- and *trans*-acting pathways [11]. Moreover, lncRNAs tend to be expressed at lower levels than protein-coding genes and display more tissue-specific expression patterns [11].

Of the many identified lncRNAs, only few have been characterized functionally [13]. A complete catalogue of lncRNAs is not yet available; nonetheless, in 2012 the GENCODE consortium published the most complete set of human lncRNAs to date [11], comprising 9277 manually annotated genes producing 14,880 transcripts. GENCODE annotate lncRNAs according to their localization regarding the nearest known protein transcripts: exonic, intronic, overlapping or intergenic [11].

To identify potential HHT therapeutic targets, further knowledge on how a disturbance of the TGF-β signalling pathway leads to HHT manifestations is needed. As lncRNAs are increasingly recognized as key regulators of gene expression we assessed the lncRNA expression in HHT tissue. Thus, the purpose of this study was to investigate the possible involvement of lncRNAs in the molecular pathogenesis of the telangiectasia formation in HHT patients. This was done by expression profiling of lncRNAs in 40 telangiectasial and 40 non-telangiectasial samples from HHT patients using microarray technology.

## Materials and Methods

### Ethics statement

This study was approved by the ethics committee of Southern Denmark (S-20090131), and the patients provided verbal and written informed consent to participate in this study in accordance with the Declaration of Helsinki.

### HHT patients

Nasal mucosal biopsy specimens of telangiectasial and non-telangiectasial tissue in pairs from HHT1 (n = 19) and HHT2 (n = 21) patients were used in this study. The nasal mucosa was anaesthetized with gauze impregnated with Lidocain phenylephrinehydrocloride. No infiltration anaesthesia was used. The samples were collected with a Weil Nasal Forceps by an experienced rhinologist and contained either macroscopicly visible telangiectasia or natural mucosa. All patients showed signs of HHT according to the Curaçao Criteria [14] and carried the familial pathogenic mutation in either *ENG* or *ACVRL1*. The 80 paired samples represented 14 different HHT1 families and 14 different HHT2 families. The familial mutations were of different types and distributed throughout the two genes (Table 1) [15]. The patients were aged 28–56 years (median age 40) and included 21 women and 19 men. Patients were diagnosed and treated in the national HHT Centre of Denmark in Odense University Hospital.

**Table 1:** Genotypes and phenotypes of the HHT patients who participated in this study.

Gene	Family No.	Nucleotid change	Amino acid change	Age	Sex	Phenotype
*ENG*	9	c.360C>A	p.Tyr120*	49	M	E,T,P,G,F
*ENG*	9	c.360C>A	p.Tyr120*	53	F	E,T,F
*ENG*	13	c.360C>A	p.Tyr120*	54	F	E,T,P,G,F
*ENG*	13	c.360C>A	p.Tyr120*	30	F	E,T,P,F
*ENG*	15	c.361-2A>G	p.?	47	F	E,T,G,F
*ENG*	15	c.361-2A>G	p.?	38	M	E,T,P,F
*ENG*	15	c.361-2A>G	p.?	40	F	E,T,F
*ENG*	24	c.360C>A	p.Tyr120*	40	F	E,T,F
*ENG*	24	c.360C>A	p.Tyr120*	46	F	E,T,P,G,F
*ENG*	37	c.360C>A	p.Tyr120*	51	M	E,T,P,F
*ENG*	38	c.360C>A	p.Tyr120*	37	F	E,T,F
*ENG*	49	c.821C>T	p.Thr274Ile	47	F	E,T,F
*ENG*	61	c.817-3T>G	p.?	49	F	E,T,F
*ENG*	65	c.1166_1168delTCT	p.Phe389del	35	F	T,P,F
*ENG*	87	c.808C>T	p.Gln270*	38	M	T,P,F
*ENG*	92	c.219+1G>T	p.?	44	M	E,T,P
*ENG*	93	c.1550_1551delTG	p.Val517Glufs*10	37	M	E,T,F
*ENG*	94	p.1582_1583del	p.Pro528Alafs*38	30	M	E,T,F
*ENG*	96	c.277C>T	p.Arg93*	46	M	E,T,P,F
*ACVRL1*	8	c.1120C>T	p.Arg374Trp	55	M	E,T,F
*ACVRL1*	8	c.1120C>T	p.Arg374Trp	28	M	E,T,F
*ACVRL1*	18	c.1468C>T	p.Gln490*	43	M	E,T,F
*ACVRL1*	18	c.1468C>T	p.Gln490*	44	F	E,T,P,F
*ACVRL1*	18	c.1468C>T	p.Gln490*	41	M	E,T,F
*ACVRL1*	20	c.430C>T	p.Arg144*	47	M	E,T
*ACVRL1*	42	c.1135G>A	p.Glu379Lys	32	F	E,T,F
*ACVRL1*	43	c.626-3C>G	p.?	38	F	E,T,F
*ACVRL1*	43	c.626-3C>G	p.?	36	F	T,F
*ACVRL1*	46	c.1013T>A	p.Val338Asp	39	M	E,T,F
*ACVRL1*	47	c.1120C>T	p.Arg374Trp	40	F	E,T,F
*ACVRL1*	56	c.1-?_1048+?del	p.0?	44	F	E,T,F
*ACVRL1*	57	c.143G>A	p.Gly48Glu	34	M	E,T,F
*ACVRL1*	57	c.143G>A	p.Gly48Glu	32	F	T,F
*ACVRL1*	67	c.266G>A	p.Cys89Tyr	24	F	E,T,F
*ACVRL1*	67	c.266G>A	p.Cys89Tyr	31	M	E,T,F
*ACVRL1*	69	c.1232G>A	p.Arg411Gln	39	M	E,T,F
*ACVRL1*	71	c.143G>A	p.Gly48Glu	44	F	E,T,F
*ACVRL1*	82	c.139_140insCG	p.Arg47Profs*8	48	M	E,T,F
*ACVRL1*	88	c.155del	p.Thr52Lysfs*2	56	F	E,T,G,F
*ACVRL1*	88	c.155del	p.Thr52Lysfs*2	50	M	E,T,F

Abbreviations: AVM, arteriovenous malformation; E, epistaxis; T, telangiectasia; P, pulmonary AVM; C, cerebral AVM; G, gastrointestinal telangiectasia/gastrointestinal bleeding; H, Hepatic AVM; F, family history. M, male; F, female.

### RNA isolation and quality

Samples were collected in RNA*later* Solution (Life Technologies) and stored at −20°C. Total RNA was isolated using the RNeasy Micro Kit according to the RNeasy® Micro Handbook (Qiagen). The quantity of RNA was assessed with the NanoDrop spectrophotometer ND-8000 (NanoDrop Technologies), and RNA quality was assessed by the Agilent 2100 Bioanalyzer (Agilent Technologies).

### Microarray labelling, hybridization, and scanning

Sample labelling and array hybridization were performed according to the Two-Color Microarray-Based Gene Expression Analysis—Low Input Quick Amp Labeling—protocol (Agilent Technologies) using the SurePrint G3 Human Gene Expression 8×60 microarray format (Agilent Technologies). Samples were labelled with Cy5 and Universal Human Reference RNA (Stratagene) was labelled with Cy3 and used as a common reference on all arrays.

### Data pre-processing and annotation of lncRNAs

Agilent Feature Extraction software v. 10.7.3.1 (Agilent technologies) was used to analyse acquired array images. Data were then within-array normalized by Loess normalization method and between-array normalized by Quantile normalization. The normalized values were used to calculate log_2_ transformed Cy5/Cy3 ratios. Replicate probes were collapsed calculating the median. Missing expression values were imputed by *k*-nearest neighbours averaging (*k* = 10). Date pre-processing was performed using the R (R Core team 2012) package *limma* [16]. Microarray data were deposited to the Gene Expression Omnibus (GSE53515).

All the 42,164 probes of the Agilent SurePrint G3 array were re-annotated using GENCODE v.16 gene annotation database (www.gencodegenes.org) [11]. The genomic coordinates of the probes in the Agilent array were matched to the genomic coordinates of the lncRNAs from the GENCODE v.16, to identify the probes covering lncRNAs. LncRNAs were included if 55 base pairs overlapped with the 60-mer array probes.

### Data analysis

Data analyses were performed using the Qlucore Omics Explorer 2.3 software (Qlucore). Differentially expressed lncRNAs comparing telangiectasial and non-telangiectasial tissue were ranked according to statistical significance determined by two-group comparison (paired t-test). This was done for the groups HHT1 and HHT2 seperately and subsequently for the total group of HHT. Multiple testing was adjusted for by the Benjamini-Hochberg method. Differentially expressed lncRNAs were chosen for further evaluation (q<0.15). Principal component analysis (PCA) and hierarchical clustering were performed in Qlucore Omics Explorer 2.3 to examine whether telangiectasial and non-telangiectasial samples could be separated.

### Genomic Regions Enrichment of Annotation

To assess the potential *cis*-regulatory effect of the differentially expressed lncRNAs in HHT, we used the Genomic Regions Enrichment of Annotations Tool (GREAT) [17]. This program has genomic coordinates as inputs and outputs nearby genes and their ontologies. It analyses the functional significance of *cis*-regulatory regions identified by localized measurements of DNA binding events across an entire genome.

### Correlation analysis

To identify potential *trans*-regulated transcripts, we assessed the correlation between the expression levels of the 10 highest ranking statistically significantly differentially expressed lncRNAs (HHT total) and every transcript present on the Agilent array by calculating Pearson's correlation coefficient. The transcripts were ranked according to the corrected p-values for the correlation coefficients; this was performed for each lncRNA of interest. Correction for multiple testing was performed using the Bonferoni correction. Manhattan plots were drawn using the Integrative Genome Viewer (IGV) version 2.3.18 [18], based on each of these correlation-sets for individual statistically significantly differentially expressed lncRNAs.

## Results

### Annotation of lncRNAs

All the 42,164 probes of the Agilent SurePrint G3 array were re-annotated using GENCODE v.16 gene annotation database (www.gencodegenes.org)[11]. We identified 5,069 probes mapping to 2,939 lncRNAs. These 5,069 probes included both intronic and exonic lncRNA probes. Of these 5,069 probes, 404 mapped to overlapping lncRNAs and mRNAs – of these, 259 probes were excluded as they mapped to overlapping mRNA exons and lncRNA introns – only probes mapping to overlapping mRNA exons and lncRNA exons were included. Further analysis was performed using the 4,810 remaining lncRNA probes, mapping to 2,811 lncRNAs, covering almost 31% of the GENCODE lncRNA dataset (Figure S1).

### Identification of differentially expressed lncRNAs in HHT telangiectasia

We detected 617 statistically significantly differentially expressed lncRNAs (q<0.05, paired t-rest) when comparing telangiectasial and non-telangiectasial samples in the total HHT group (80 paired samples), of which 42 were highly statistically significant (q<0.001). Of the 42 differentially expressed lncRNAs (listed in Table 2), 16 were upregulated and 26 downregulated. None of these 42 lncRNAs are characterized functionally.

**Table 2:** Top 42 long non-coding RNAs (q<0.001) differentially expressed in HHT telangiectasia.

Ensembl gene ID	gencode.v16_GenomicCoordinates	q-value (FDR)	Fold change	Gene biotype	HGNC symbol
ENSG00000249772.1	chr5:80409204-80410671_R	1.83E-06	0.86	antisense	-
ENSG00000230544.1	chr13:114586640-114588308_F	1.50E-05	0.88	lincRNA	LINC00453
ENSG00000215231.3	chr5:5034472-5070117_F	1.50E-05	0.88	lincRNA	-
ENSG00000237548.1	chr9:124646915-124725998_R	1.50E-05	0.86	processed_transcript	TTLL11-IT1
ENSG00000263753.1	chr18:5232875-5246507_F	0.00011	0.90	lincRNA	LINC00667
ENSG00000231133.1	chr20:61727150-61733631_R	0.00014	0.90	processed_transcript	HAR1B
ENSG00000256218.1	chr12:5475214-5476940_R	0.00015	0.90	lincRNA	-
ENSG00000241269.1, ENSG00000188365.3	chr7:5459458-5462753_F	0.00022	0.85	antisense	-
ENSG00000226496.1	chr21:42513427-42520060_R	0.00023	1.14	antisense	LINC00323
ENSG00000248176.1	chr4:29119930-29204392_F	0.00039	0.89	lincRNA	-
ENSG00000259484.1	chr15:57151866-57210697_R	0.00039	0.86	processed_transcript	-
ENSG00000206129.3	chr18:53670844-53858493_R	0.00042	1.11	lincRNA	-
ENSG00000235285.1	chr13:44720606-44732358_R	0.00042	0.90	sense_intronic	SMIM2-IT1
ENSG00000147753.5	chrY:6317509-6325947_F	0.00042	1.13	lincRNA	TTTY7
ENSG00000240453.1	chr1:745489-753092_R	0.00042	0.84	processed_transcript	-
ENSG00000259150.1	chr15:26360960-26378184_F	0.00042	1.09	lincRNA	LINC00929
ENSG00000255471.1*	chr11:86603256-86636079_R	0.00045	0.81	antisense	-
ENSG00000237036.3	chr10:31596646-31608810_R	0.00056	1.11	antisense	-
ENSG00000233154.1	chr1:116966346-117021464_R	0.00065	0.91	lincRNA	-
ENSG00000197251.3	chr6:33553883-33561115_R	0.00068	1.10	antisense	LINC00336
ENSG00000248176.1	chr4:29119930-29204392_F	0.00073	0.92	lincRNA	-
ENSG00000254154.3	chr1:177897923-178007142_R	0.00073	0.88	processed_transcript	-
ENSG00000215374.4	chr8:7159133-7212876_R	0.00073	0.90	processed_transcript	-
ENSG00000196096.3	chr2:214141276-214148929_R	0.00073	0.92	processed_transcript	-
ENSG00000259758.1	chr8:141530255-141539600_R	0.00073	1.13	lincRNA	CASC7
ENSG00000264772.1, ENSG00000265500.1	chr17:7466604-7485342_F	0.00074	1.26	processed_transcript	-
ENSG00000229563.1	chrX:45364633-45489447_F	0.00076	1.10	processed_transcript	-
ENSG00000203325.3	chr1:32517892-32539075_R	0.00076	0.88	antisense	-
ENSG00000232956.3*	chr7:45022622-45026560_R	0.00076	1.20	lincRNA	-
ENSG00000232021.2*	chr4:109088681-109177992_F	0.00076	0.90	processed_transcript	LEF1-AS1
ENSG00000250195.1	chr4:139741108-139933800_R	0.00076	1.21	antisense	-
ENSG00000250608.1	chr3:131043936-131100319_R	0.00076	1.11	processed_transcript	-
ENSG00000266952.1	chr18:61880317-61927290_R	0.00078	0.91	lincRNA	-
ENSG00000259334.1	chr14:24391456-24403777_R	0.00079	1.11	lincRNA	LINC00596
ENSG00000249364.1	chr5:66675206-67101066_F	0.00079	0.91	processed_transcript	-
ENSG00000231185.2*	chr5:141704858-142051566_F	0.00086	0.93	processed_transcript	-
ENSG00000232046.1	chr2:66801162-66957289_F	0.00086	0.91	processed_transcript	-
ENSG00000230133.1	chr20:24180403-24205224_F	0.00086	0.90	lincRNA	-
ENSG00000215808.2	chr1:238643684-238649323_R	0.00090	1.11	processed_transcript	-
ENSG00000135253.9	chr7:128502505-128550773_R	0.00094	1.08	processed_transcript	KCP
ENSG00000233251.3	chr2:56400669-56412905_R	0.00094	1.09	processed_transcript	-
ENSG00000245910.3	chr8:67833919-67838633_R	0.0010	0.85	processed_transcript	SNHG6

*Are part of the lncRNAs identified by GREAT analysis.

**Figure 1:**
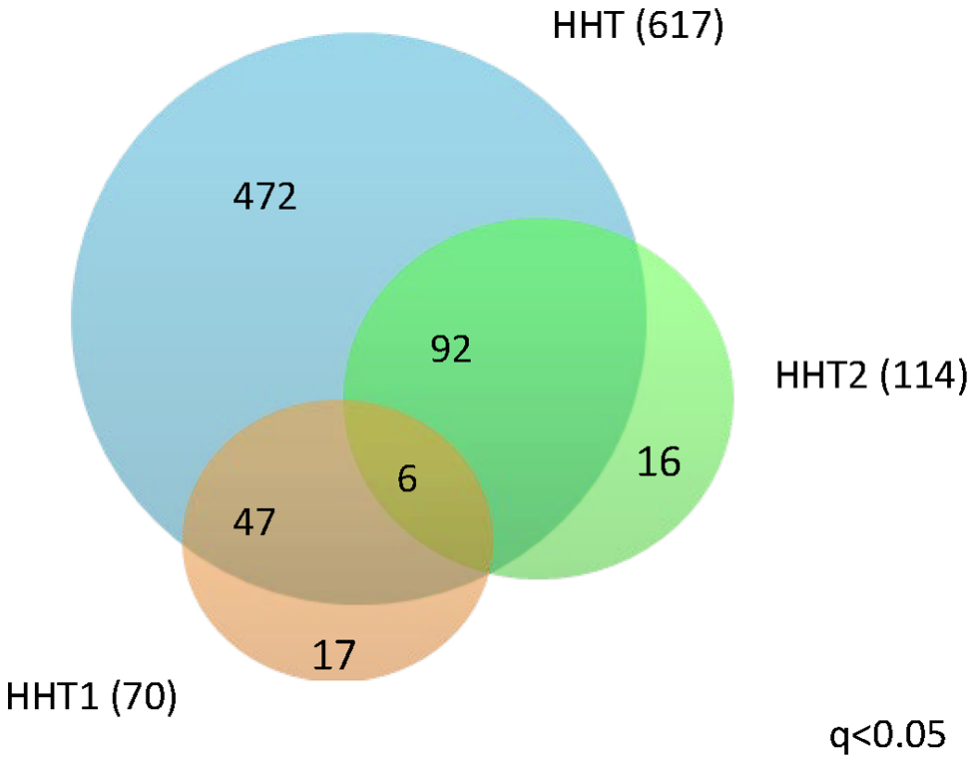
Venn diagram. The number of statistically significantly differentially expressed long non-coding RNAs in the three groups (q<0.05). The numbers in the overlapped areas indicate the lncRNAs that are differentially expressed in the multiple groups.

Analysis of the HHT1 samples resulted in 70 differentially expressed lncRNAs (q<0.05, paired t-test) when comparing telangiectasial and non-telangiectasial samples, and in HHT2 analysis resulted in 114 differentially expressed lncRNAs (q<0.05, paired t-test). The number of common lncRNAs (q<0.05) in the three groups is rather small as shown in Figure 1.

The complete results of the paired t-tests in HHT and the subgroups HHT1 and HHT2 are provided in Table S1, S2, and S3.

### Principal component analysis (PCA) and hierarchical clustering

**Figure 2:**
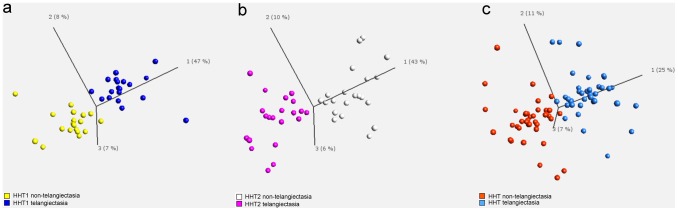
Principal component analysis (PCA) of the three groups. a. PCA applied to the differentially expressed long non-coding RNAs (lncRNAs)(paired t-test, q<0.15) revealed a clear separation of the telangiectasial and non-telangiectasial samples, regarding HHT1. b. PCA applied to the differentially expressed lncRNAs (paired t-test, q<0.15) revealed a clear separation of the telangiectasial and non-telangiectasial samples, regarding HHT2. c. PCA applied to the differentially expressed lncRNAs (paired t-test, q<0.15) revealed a clear separation of the telangiectasial and non-telangiectasial samples in the total group of HHT. There is no clustering into subgroups.

PCA applied to the differentially expressed lncRNAs (q<0.15) revealed a clear separation of the telangiectasial and non-telangiectasial samples, regarding HHT1 (Figure 2a), HHT2 (Figure 2b) and HHT (Figure 2c). No statistically significant difference in expression values between HHT1 telangiectasial tissue and HHT2 telangiectasial tissue was observed. Even so, the data suggest that there is a minor difference as the comparison and grouping of the subgroups introduce more variation in the PCA plots (Figures 2a and 2b compared with Figure 2c).

**Figure 3:**
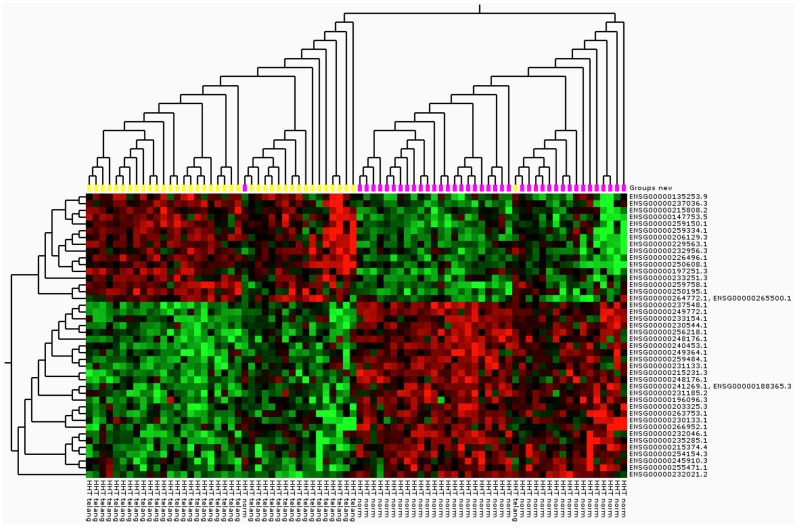
Hierarchical clustering HHT. Hierarchical clustering for the 80 HHT paired samples comparing the expression in telangiectasial (yellow) and non-telangiectasial (pink) tissue, using the top variant lncRNAs (n = 42)(q<0.001). In the heat map rows correspond to long non-coding RNAs (lncRNAs) and columns to samples. Red indicates elevated expression, green indicates reduced expression. In the 42 differentially expressed lncRNAs; 16 are up-regulated and 26 are down-regulated.

Hierarchical clustering: amongst the differentially expressed lncRNAs (q<0.001) in the HHT group both up- and down-regulation was observed (Figure 3). Hierarchical clustering for HHT1 and HHT2 is shown in Figure S6 and S7.

### Genomic Regions Enrichment of Annotation

#### HHT1

**Figure 4:**
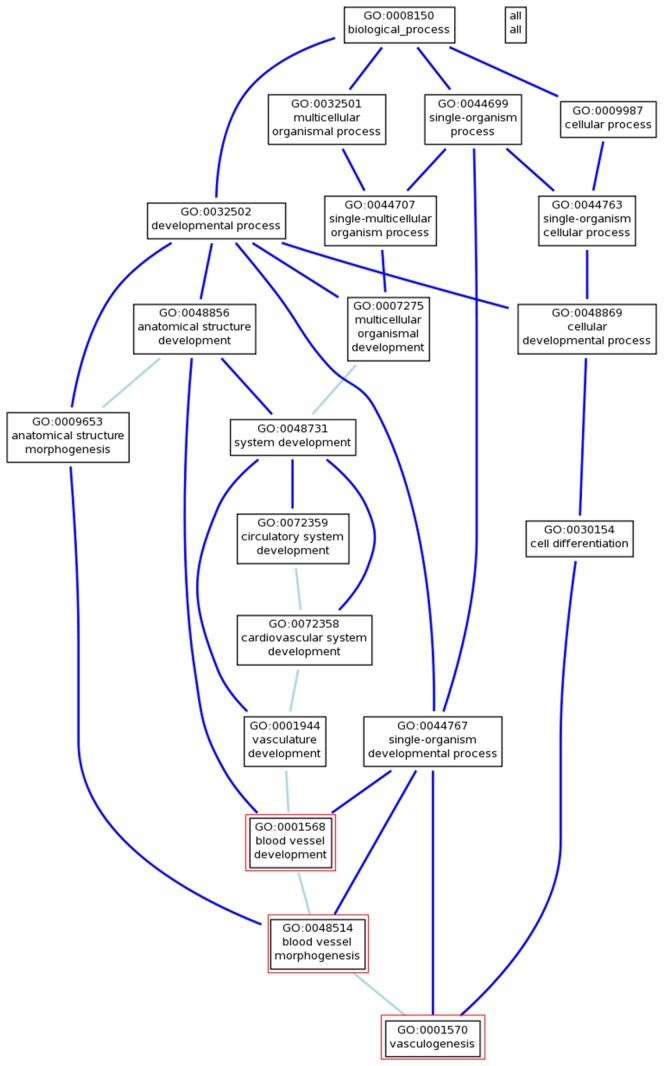
Gene Ontology terms tree. Graphical view of the gene ontology (GO) terms tree showing part of the GO terms in the ontology biological process and their connections. Our three GO terms; blood vessel development, blood vessel development, and vasculogenesis (in red boxes), are the more narrow and specific terms in the tree and overlap by a number of genes.

The differentially expressed lncRNAs with q<0.15 (n = 617) were loaded into GREAT. Of these, 31% map within 50 kilobases (kb) of an annotated gene, and 89% map within 500 kb of a gene (Figure S2). Gene ontology [19] (www.geneontology.org) analysis, performed in GREAT, of the 617 neighbouring genomic regions (826 genes) is seen in Table 3. The ontologies of those loci concerned six gene ontology (GO) terms (binomial false discovery rate (FDR) q-value<0.001), of which three were considered key terms as they are central to HHT pathogenesis: blood vessel morphogenesis (GO:0001568, 3747 gene products), blood vessel development (GO:0001568, 3747 gene products) and vasculogenesis (Synonym: Vascular morphogenesis, GO:0001570, 557 gene products). Table 4 (and Tables S4–S6 in File S1) lists the genes involved in the three GO terms and their relative positions. In the GO terms tree (Figure 4) the close connection and overlap of the three mentioned GO terms can be seen.

**Table 3:** Gene ontology (GO) analysis of 617 genomic regions (826 genes) located nearby differentially expressed long non-coding RNAs in the HHT1 group.

Ontology	Term name	Term ID	Binom FDR Q-value	Binom fold enrichment	Hyper FDR Q-value	Hyper fold enrichment
GO Biological Process	blood vessel morphogenesis	GO:0048514	9.21e-7	2.29	4.59602e-5	2.72
GO Biological Process	blood vessel development	GO:0001568	6.81e-6	2.07	5.89303e-5	2.52
GO Biological Process	immune system development	GO:0002520	7.30e-5	2.06	5.60561e-3	2.06
GO Biological Process	hemopoietic or lymphoid organ development	GO:0048534	1.74e-4	2.04	1.14257e-2	2.04
GO Biological Process	hemopoiesis	GO:0030097	4.03e-4	2.06	1.65733e-2	2.06
GO Biological Process	vasculogenesis	GO:0001570	6.00e-4	3.43	5.61511e-4	4.90

**Table 4:** Overlap between gene-genomic regions association tables for the different GO terms.

GO Blood vessel morphogenesis (HHT1)	GO Blood vessel development (HHT1)	GO Vasculogenesis (HHT1)	GO Vasculogenesis (HHT2)	GO Vasculogenesis (HHT)	GO Blood vessel morphogenesis (HHT)	Gene located on Chromosome	Foldchange (ENSG)
*AMOT*	*AMOT*	*AMOT*				2	1.09
					*ANGPT2*	8	1.13
*APOB*	*APOB*				*APOB*	14	1.06–1.07
*BMP4*	*BMP4*				*BMP4*	14	0.92–0.93
***CAV1***	***CAV1***	***CAV1***	***CAV1***	***CAV1***	***CAV1***	7	**1.01–1.18**
***CCM2***	***CCM2***	***CCM2***	***CCM2***	***CCM2***	***CCM2***	7	**1.16–1.25**
	*CDH5*					16	0.89
			*CITED2*	*CITED2*	*CITED2*	6	0.92–0.93
*CXCL12*	*CXCL12*				*CXCL12*	10	0.89–0.92
*CXCR4*	*CXCR4*					2	1.10
*CYP1B1*	*CYP1B1*				*CYP1B1*	2	0.89–0.90
*FGF1*	*FGF1*				*FGF1*	5	0.09–1.16
*FN1*	*FN1*				*FN1*	2	0.88–1.22
***FOXF1***	***FOXF1***	***FOXF1***	***FOXF1***	***FOXF1***	***FOXF1***	16	**1.06–1.09**
	*FOXO1*					13	1.11
***FZD4***	***FZD4***	***FZD4***	***FZD4***	***FZD4***	***FZD4***	11	**0.65–1.22**
*GREM1*	*GREM1*					15	1.09
*HDAC7*	*HDAC7*	*HDAC7*		*HDAC7*	*HDAC7*	12	0.91–0.92
*HES1*	*HES1*				*HES1*	3	0.91–1.14
					*ITGB1*	10	1.09
*ITGA5*	*ITGA5*					12	1.19–1.20
*JAG1*	*JAG1*					20	1.27
*JAM3*	*JAM3*					11	0.95
*LEF1*	*LEF1*				*LEF1*	4	0.87–0.90
	*MEF2C*					5	1.09–1.11
*MEIS1*	*MEIS1*				*MEIS1*	2	0.57–1.14
*NOX5*	*NOX5*					15	0.93
					*NR2F2*	15	0.92
					*NRP1*	10	1.09
*NRXN1*	*NRXN1*				*NRXN1*	2	0.94–0.95
*PRKX*	*PRKX*					X	1.08
*PROK2*	*PROK2*				*PROK2*	3	1.08–1.20
***PRSS23***	***PRSS23***	***PRSS23***	***PRSS23***	***PRSS23***	***PRSS23***	11	**0.65–0.93**
***RASA1***	***RASA1***	***RASA1***	***RASA1***	***RASA1***	***RASA1***	5	**0.92–1.16**
			*SHH*	*SHH*	*SHH*	7	0.88–1.09
*SOX4*	*SOX4*				*SOX4*	6	0.92–1.12
*STAB2*	*STAB2*				*STAB2*	12	0.92–0.94
*T*	*T*	*T*	*T*			6	1.16–1.28
*TGFB2*	*TGFB2*				*TGFB2*	1	1.08–1.13
*THBS1*	*THBS1*				*THBS1*	15	0.93–1.17
***TIPARP***	***TIPARP***	***TIPARP***	***TIPARP***	***TIPARP***	***TIPARP***	3	**0.84–1.08**
			*VEGFA*	*VEGFA*	*VEGFA*	6	1.07–1.24
*WARS*	*WARS*					14	0.92
	*WNT2*					7	0.93
*WNT7A*	*WNT7A*	*WNT7A*		*WNT7A*	*WNT7A*	3	0.91–1.10
			*WNT7B*			22	0.90
*ZC3H12A*	*ZC3H12A*					1	0.92
*ZFPM2*	*ZFPM2*	*ZFPM2*	*ZFPM2*	*ZFPM2*	*ZFPM2*	8	1.13–1.23
***ZMIZ1***	***ZMIZ1***	***ZMIZ1***	***ZMIZ1***	***ZMIZ1***	***ZMIZ1***	10	**0.01–1.11**

q-values can be seen of tables S4–S9. Genes that are present for all six GO–terms are highlighted in bold.

#### HHT2

The differentially expressed lncRNAs with q<0.15 (n = 640) were loaded into GREAT. Of these, 31% map within 50 kilobases (kb) of an annotated gene, and 89% map within 500 kb of a gene (Figure S3). Gene ontology analysis of the 640 neighbouring genomic regions (887 genes) is seen in Table 5. The ontologies of those loci concerned five GO terms (binomial FDR q-value<0.001), of which especially vasculogenesis was interesting. Table 4 (and Table S7 in File S1) lists the genes involved in this GO term and their relative positions.

**Table 5:** Gene ontology (GO) analysis of 640 genomic regions (887 genes) located nearby differentially expressed long non-coding RNAs in the HHT2 group.

Ontology	Term name	Term ID	Binom FDR Q-value	Binom fold enrichment	Hyper FDR Q-value	Hyper fold enrichment
GO Biological Process	vasculogenesis	GO:0001570	1.90e-5	3.80	6.20e-5	4.91
GO Biological Process	positive regulation of neuron differentiation	GO:0045666	4.95e-5	3.13	4.51e-4	4.41
GO Biological Process	regulation of mesenchymal cell proliferation	GO:0010464	3.90e-4	3.28	3.45e-3	5.00
GO Biological Process	regulation of DNA binding	GO:0051101	7.35e-4	3.24	2.21e-5	5.00
GO Biological Process	regulation of binding	GO:0051098	8.32e-4	2.43	1.13e-5	3.38

#### HHT

The differentially expressed lncRNAs with q<0.05 (n = 617) were loaded into GREAT. A more stringent cut-off of q<0.05 was applied here, as the sample size was larger. Of these lncRNAs, 31% map within 50 kilobases (kb) of an annotated gene, and 90% map within 500 kb of a gene (Figure S4). Gene ontology analysis of the 617 neighbouring genomic regions (837 genes) is seen in Table 6. Applying a cut-off of q<0.001 resulted in three GO terms: vasculogenesis, lactation and blood vessel morphogenesis. Table 4 (and Tables S8 and S9 in File S1) lists the genes involved in the GO terms ‘vasculogenesis’ and ‘blood vessel morphogenesis’ and their relative positions.

**Table 6:** Gene ontology (GO) analysis of 617 genomic regions (837 genes) located nearby differentially expressed long non-coding RNAs in the total HHT group.

Ontology	Term name	Term ID	Binom FDR Q-value	Binom fold enrichment	Hyper FDR Q-value	Hyper fold enrichment
GO biological process	vasculogenesis	GO:0001570	1.71e-5	3.94	4.92e-5	5.21
GO biological process	lactation	GO:0007595	2.06e-5	6.51	9.57e-3	4.85
GO biological process	blood vessel morphogenesis	GO:0048514	1.92e-4	2.01	4.06e-4	2.40

By GREAT analysis, 63 lncRNAs were identified, which is equal to 10.2% of the differentially expressed lncRNAs in the HHT group (n = 617, q<0.05). Concordingly, 89.8% of statistically significantly differentially expressed lncRNAs in the HHT group are not part of the three selected statistically significant GO terms in GREAT.

### Correlation analysis

**Figure 5:**
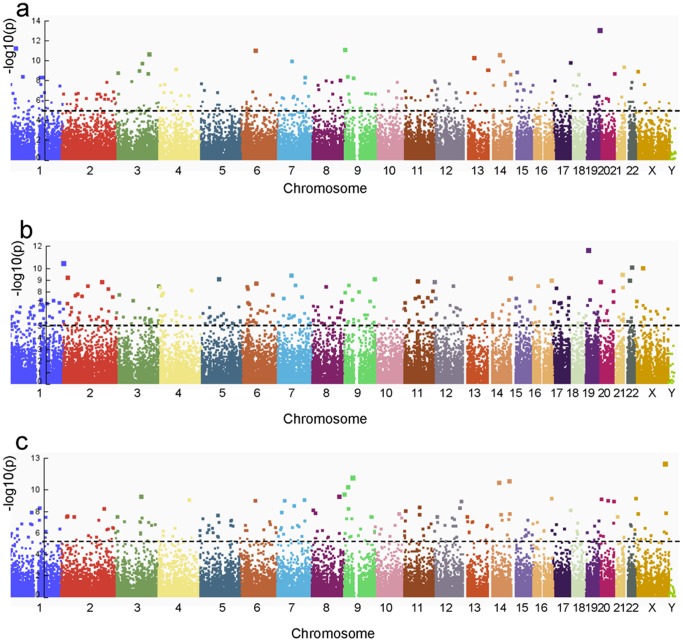
Manhattan plots. Manhattan plots showing significance of correlation between the three top statistically significantly differentially expressed long non-coding RNAs' (lncRNAs) expression and expression of all other genes at the microarray. The negative logarithm (-log_10_) p-values of the Pearson correlation were plotted across chromosomes. The Bonferoni-corrected significance level is indicated by the dashed line (p<0.05). a. ENSG00000249772.1 (Chromosome 5) has 144 statistically significantly correlated transcripts, of which 62.5% are other lncRNAs. b. ENSG00000230544.1 (Chromosome 13) has 178 statistically significantly correlated transcripts, of which 61% are other lncRNAs. c. ENSG00000215231.3 (Chromosome 5) has 158 statistically significantly correlated transcripts, of which 63% are other lncRNAs. For comparison, only 11% of the probes across the microarray map to lncRNAs.

To identify potential *trans*-regulated transcripts, we assessed the correlation between the expression levels of the 10 highest ranking differentially expressed lncRNAs in the Hereditary Haemorrhagic Telangiectasia (HHT) total group and all transcripts present on the Agilent array, by calculating Person's correlation coefficient. The Manhattan plots drawn on this basis showed that several statistically significant correlations were present for each lncRNA in question (40-3725 correlations, p<0.05 (Bonferoni-corrected)(data not shown)), and that the correlating transcripts were mapping to many different chromosomes (Figure 5 shows the top three ranking lncRNAs). More than half the correlated transcripts were lncRNAs (data not shown). In the cases of multiple lncRNAs neighbouring a gene you see a clear correlation of foldchange/direction; meaning that if one lncRNA neighbouring a gene is upregulated, in most cases (37/49) so are the other(s) (Table 4).

## Discussion

To our knowledge, this is the first study to assess the regulatory effects of long noncoding RNAs (lncRNAs) in HHT affected tissue. Using microarray technology, we identified lncRNAs that are statistically significantly differentially expressed in HHT telangiectasial tissue compared with HHT non-telangiectasial nasal tissue. Using GREAT, a tool which assumes *cis*-regulation, we also showed that those lncRNAs are enriched for genomic loci involved in key pathways concerning HHT. The comparison of expression profiles between HHT telangiectasial and HHT non-telangiectasial nasal tissue clearly showed the differential expression of a substantial number of lncRNAs. Principal component analysis (PCA) and hierarchical clustering were able to discriminate between HHT telangiectasial and HHT non-telangiectasial nasal tissue, demonstrating an HHT telangiectasial profile. However, gene expression profiling did not allow us to discriminate between HHT1 and HHT2, possibly because of the size of the study and lack of statistical power. Nonetheless, HHT1 and HHT2 had somewhat different groups of differentially expressed lncRNAs with a smaller number of common lncRNAs, which may be due to the minor phenotypical and pathway differences of HHT1 and HHT2.

The lncRNA list used in this study was retrieved from the GENCODE v.16 dataset, which uses a combination of manual annotation, computational analysis and targeted experimental validation and is the largest catalogue of human lncRNAs to date. It was used in order to retrieve optimal lncRNA annotation. To minimize the number of probes mapping only or mostly to mRNAs, we filtered out probes mapping to overlapping mRNA exons and lncRNA introns.

LncRNAs appear to control expression of protein-coding genes through both *cis*- and *trans*-acting pathways. As *cis*-regulation is reported to be more pronounced for lncRNAs [11], we chose to use GREAT for further analysis of our data. GREAT is a tool that can be used to relate the lncRNA transcriptome to biology using well-known mRNA function and gene ontology (GO) terms. Using GREAT, three interesting and statistically significant GO terms were the result: blood vessel morphogenesis, blood vessel development, and vasculogenesis. All three were located inside the top 6 resulting GO terms. Blood vessel morphogenesis is defined as: ‘The process in which the anatomical structures of blood vessels are generated and organized. The blood vessel is the vasculature carrying blood’. Blood vessel development is defined as: ‘The process whose specific outcome is the progression of a blood vessel over time, from its formation to the mature structure’; and vasculogenesis: ‘The differentiation of endothelial cells from progenitor cells during blood vessel development, and the de novo formation of blood vessels and tubes’. These GO term results are interesting as HHT manifestations are thought to result from imbalanced angiogenesis. The results may point to a causal effect of lncRNAs regulating mRNAs central to vasculogenesis, angiogenesis, and perhaps even HHT telangiectasia formation.

In most of the cases of multiple lncRNAs neighbouring a gene, a clear correlation of direction was observed. However, a correlation of direction was not present in all cases, which could indicate that a more fine-tuned regulation is being played out. Each GO term involved both up- and downregulated lncRNAs, which may suggest local *cis*-regulation at multiple sites.

The three GO terms contain repeatedly mentioned genes. Accordingly, that indicates a central group of genes that seems to be *cis*-regulated by differentially expressed lncRNAs. This central group of genes primarily involves: *CAV1, CCM2, FOXF1, FZD4, PRSS23, RASA1, SMO, TIPARP, ZFPM2* and *ZMIZ1*. Some of these are genes known to cause vascular malformation disorders, such as HHT: *CCM2* causes cerebral cavernous malformation type 2 [20] and *RASA1* causes capillary malformation-arteriovenous malformation syndrome (CM-AVM) [21].

Another approach in the data analysis was the correlation study. The correlations between the expression levels of the 10 highest ranking differentially expressed lncRNAs in the HHT total group and every transcript present in the Agilent microarray were assessed by calculating Pearson's correlation coefficient. Multiple statistically significant correlations, mapping to many different chromosomes, were present for each lncRNA in question. Hence, the correlation analyses may indicate that multiple *trans*-regulative mechanisms are present. More than half the correlated transcripts are lncRNAs and, as these tend to be more tissue-specific than mRNAs, it could be that they are all co-regulated. Nevertheless, these results suggest only a certain degree of whole-genome correlation, and this may not be associated to the HHT phenotype at all.

In 2007, two gene expression microarray studies were published describing HHT samples. *Thomas et al*. [22] studied cultured human umbilical vein endothelial cells from seven newborns carrying the familial HHT mutation, using human umbilical vein endothelial cells from three healthy newborns as controls. *Fernandez-Lopez et al*. [23] studied blood outgrowth endothelial cells from three HHT patients using blood outgrowth endothelial cells from healthy donors as controls. Neither of the two papers described non-coding RNA, and their raw data have not been made available for re-annotation and data analysis in order to compare with our results.

The strength of our study is that affected HHT tissue was analysed in a paired design with samples from a relatively large number of patients. This resulted in a large number of differently expressed lncRNAs, even though we applied a strict cut-off. GREAT is a useful tool to relate lncRNAs to known biology, but it is limited to *cis*-regulation. Of the 617 aberrantly expressed lncRNAs in the HHT group, only 63 were identified in relevant GO terms by GREAT, indicating that regulative mechanisms other than *cis*-regulation may be important. Future studies are needed to validate the results of our study and to contribute knowledge about specific lncRNA functionality and both *cis*- and *trans*-regulatory mechanisms.

In summary, our study identified lncRNAs that are aberrantly expressed in HHT telangiectasia and indicates that lncRNAs may contribute to regulate protein-coding loci in HHT, suggesting that the lncRNA component of the transcriptome deserves more attention in HHT research. Thus, a deeper understanding of lncRNAs and their role in telangiectasia formation possesses potential for discovering therapeutic targets and for identifying new biomarkers.

## Supporting Information

Figure S1 **Comparison of Agilent probe genomic coordinates and Gencode v.16 long non-coding RNAs genomic coordinates.**
(TIFF)

Figure S2 **Relative orientation and distance (kb) to the nearest transcriptional start site (TSS) of all differentially expressed long non-coding RNAs (q<0.15) in the HHT1 group.**
(TIFF)

Figure S3 **Relative orientation and distance (kb) to the nearest transcriptional start site (TSS) of all differentially expressed long non-coding RNAs (q<0.15) in the HHT2 group.**
(TIFF)

Figure S4 **Relative orientation and distance (kb) to the nearest transcriptional start site (TSS) of all differentially expressed long non-coding RNAs (q<0.15) in the HHT total group.**
(TIFF)

Figure S5 **Venn diagram: Overlap of long non-coding RNAs from the subgroups that are loaded into GREAT.**
(TIFF)

Figure S6 **Hierarchical clustering HHT1**.
(TIFF)

Figure S7 **Hierarchical clustering HHT2**.
(TIFF)

Table S1 **Dataset - paired t-test HHT1.**
(XLS)

Table S2 **Dataset - paired t-test HHT2.**
(XLS)

Table S3 **Dataset - paired t-test HHT.**
(XLS)

File S1 **Contains the Tables S4–S9.** Table S4. Gene-genomic regions association table for GO term ‘blood vessel morphogenesis’ (HHT1); Table S5. Gene-genomic regions association table for the GO term ‘blood vessel development’ (HHT1); Table S6. Gene-genomic regions association table for GO term ‘vasculogenesis’ (HHT1); Table S7. Gene-genomic regions association table for GO term ‘vasculogenesis’ (HHT2); Table S8. Gene-genomic regions association table for GO term ‘vasculogenesis’ (HHT); and Table S9. Gene-genomic regions association table for GO term ‘blood vessel morphogenesis’ (HHT).
(DOCX)
