# Supplementary figures and images for: Long Non-Coding RNA Expression Profiles in Hereditary Haemorrhagic Telangiectasia
Source: PLoS One. 2014 Mar 6;9(3):e90272. doi: 10.1371/journal.pone.0090272 (PMC3946172; doi:10.1371/journal.pone.0090272)

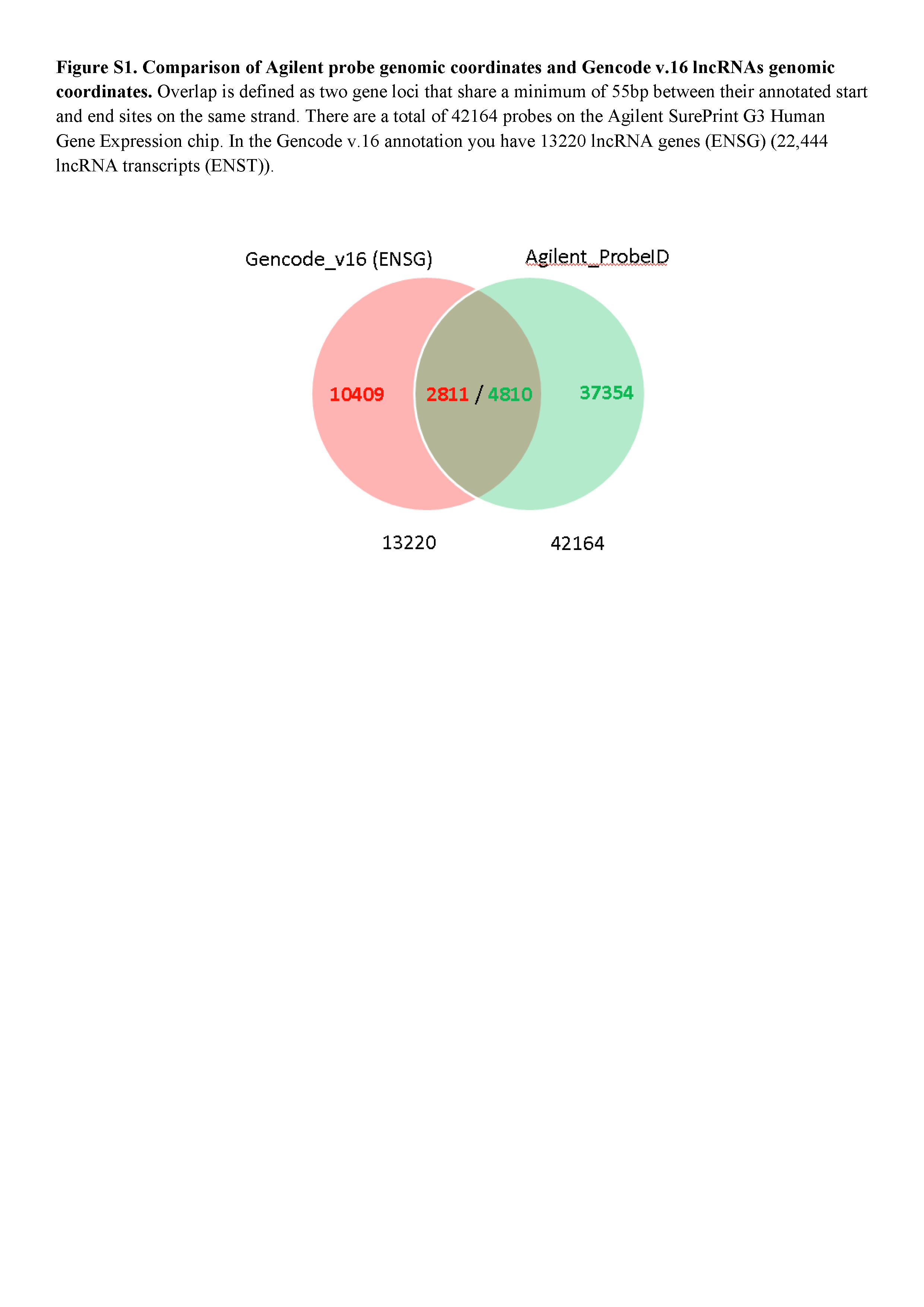

Supplement: Figure S1 — Comparison of Agilent probe genomic coordinates and Gencode v.16 long non-coding RNAs genomic coordinates. (TIFF) [file pone.0090272.s001.tif]

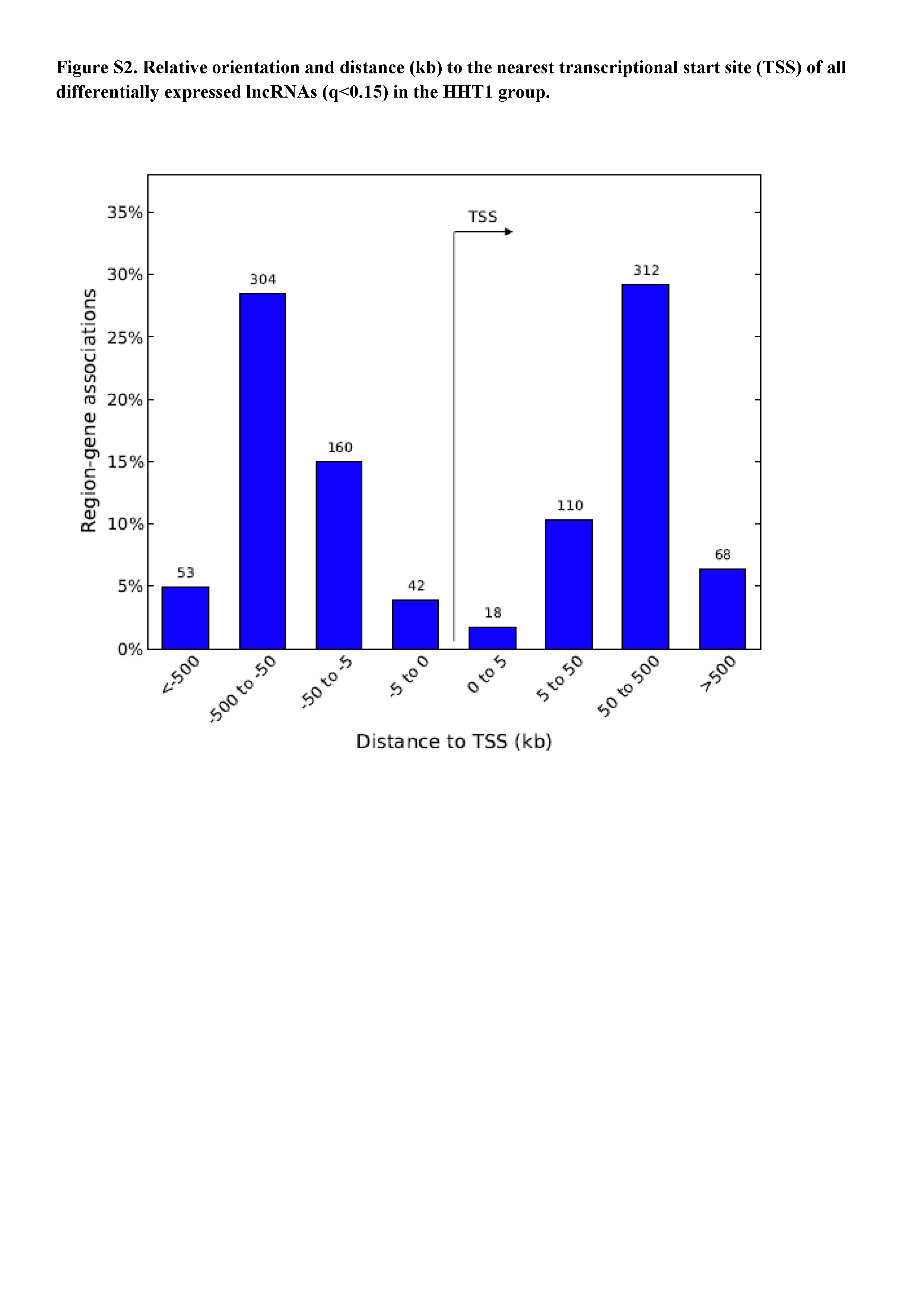

Supplement: Figure S2 — Relative orientation and distance (kb) to the nearest transcriptional start site (TSS) of all differentially expressed long non-coding RNAs (q<0.15) in the HHT1 group. (TIFF) [file pone.0090272.s002.tif]

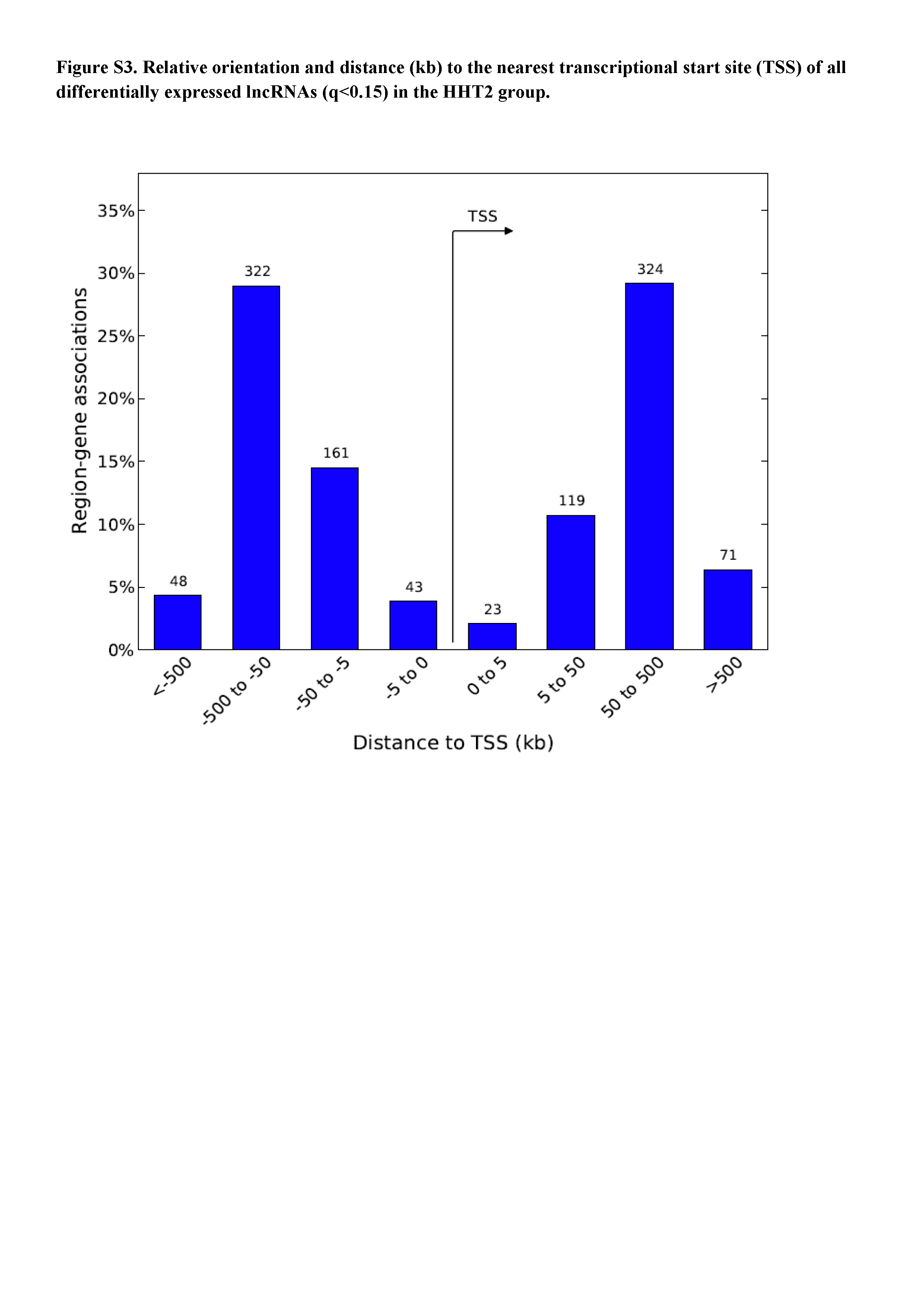

Supplement: Figure S3 — Relative orientation and distance (kb) to the nearest transcriptional start site (TSS) of all differentially expressed long non-coding RNAs (q<0.15) in the HHT2 group. (TIFF) [file pone.0090272.s003.tif]

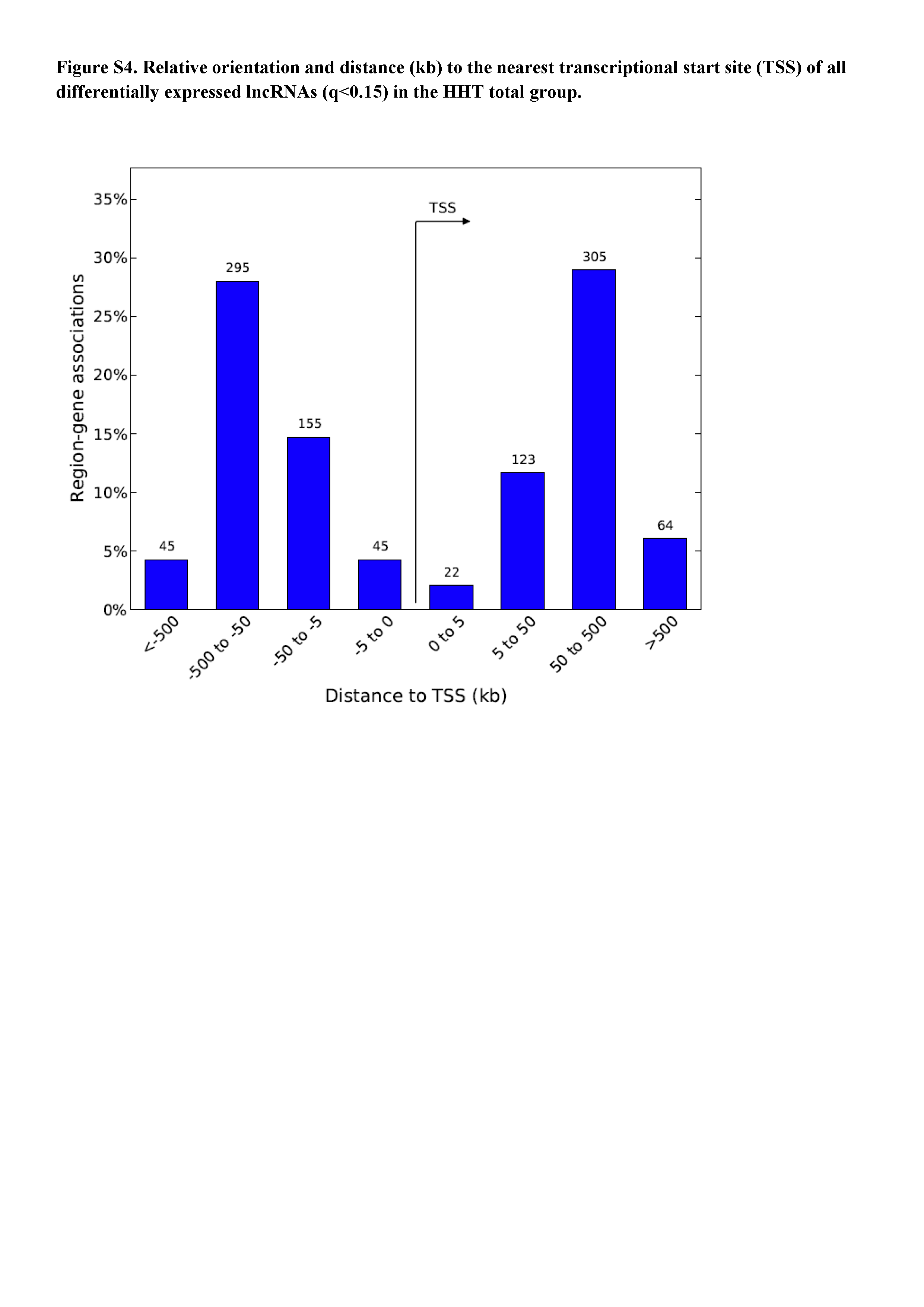

Supplement: Figure S4 — Relative orientation and distance (kb) to the nearest transcriptional start site (TSS) of all differentially expressed long non-coding RNAs (q<0.15) in the HHT total group. (TIFF) [file pone.0090272.s004.tif]

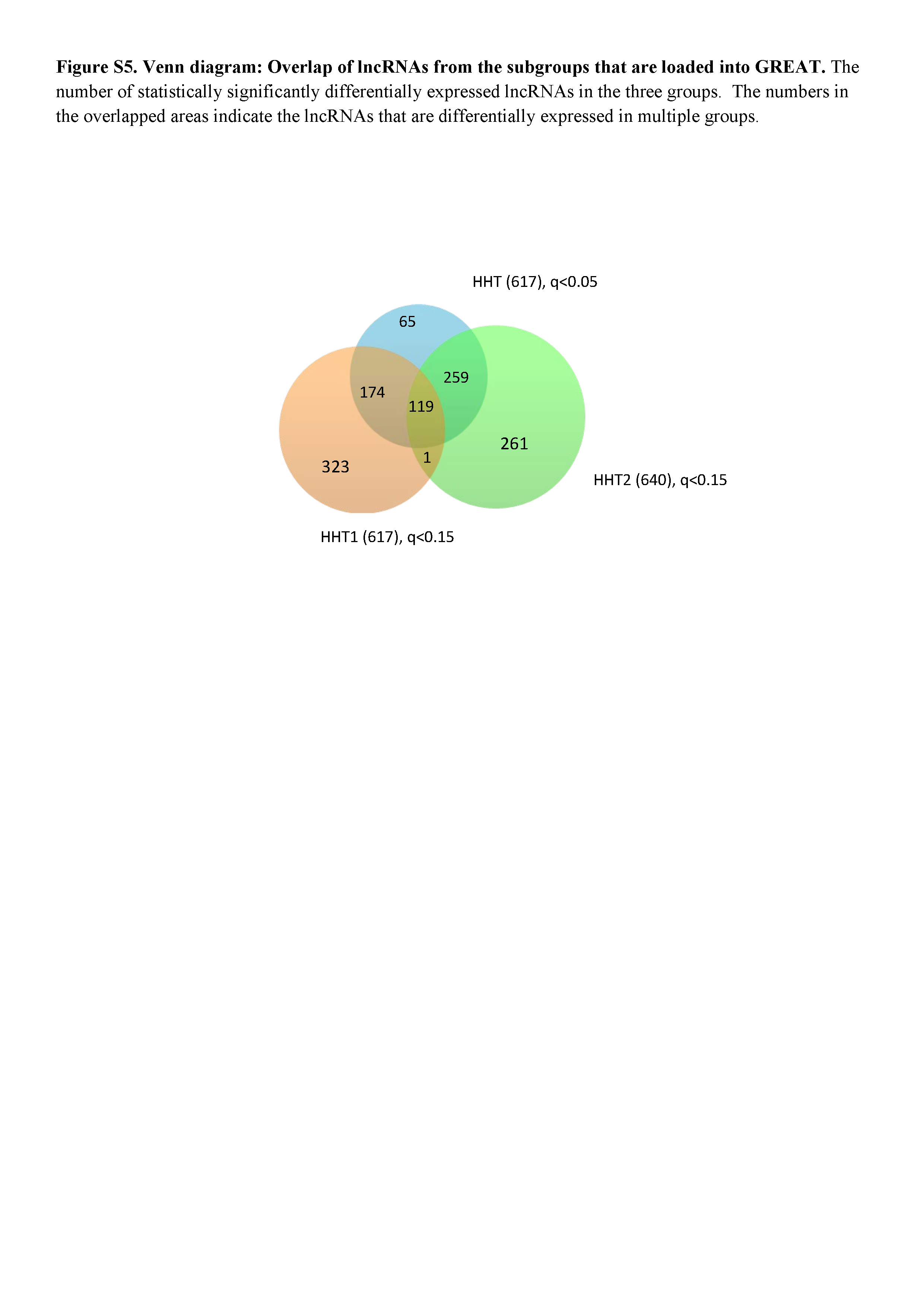

Supplement: Figure S5 — Venn diagram: Overlap of long non-coding RNAs from the subgroups that are loaded into GREAT. (TIFF) [file pone.0090272.s005.tif]

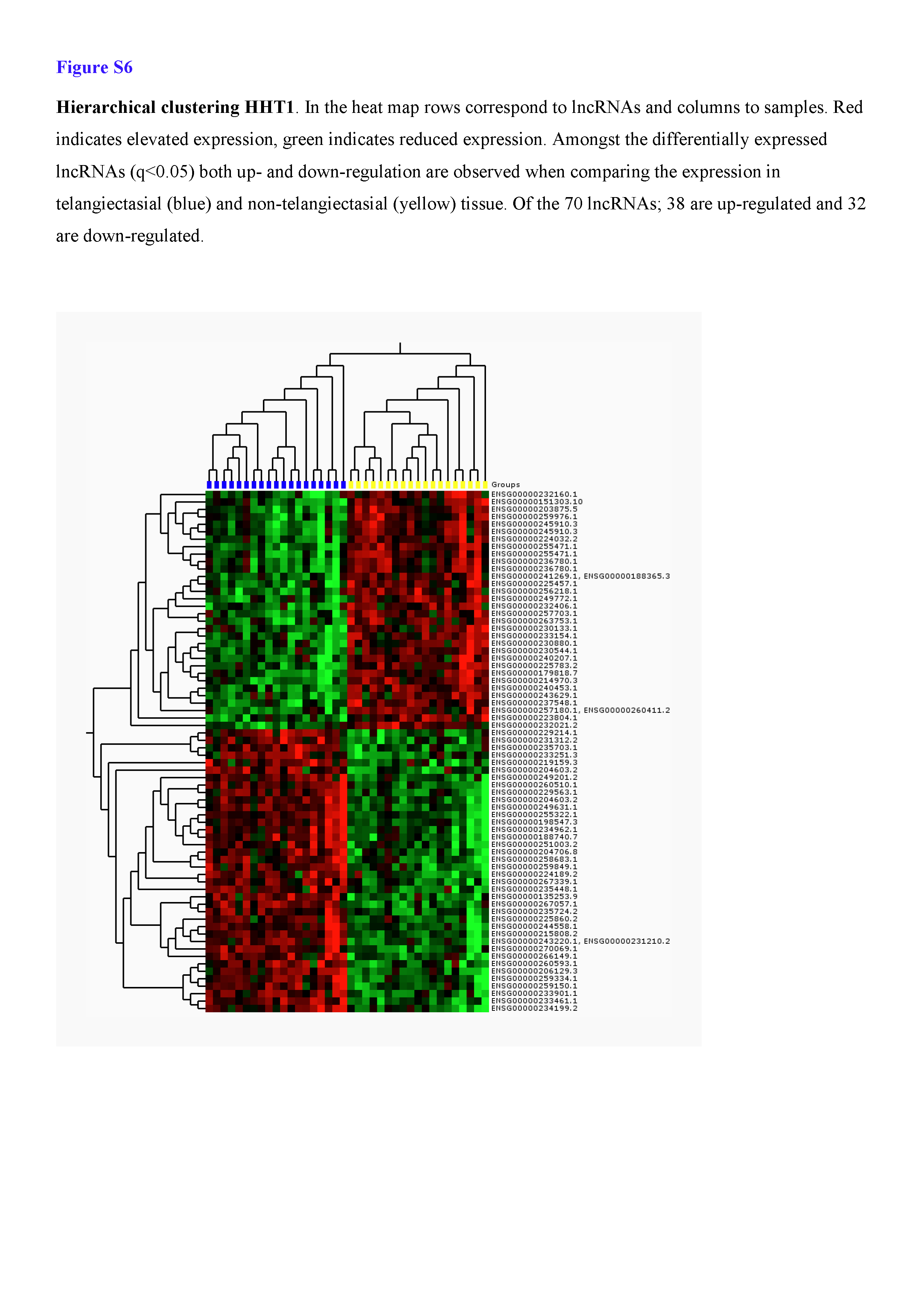

Supplement: Figure S6 — Hierarchical clustering HHT1. (TIFF) [file pone.0090272.s006.tif]

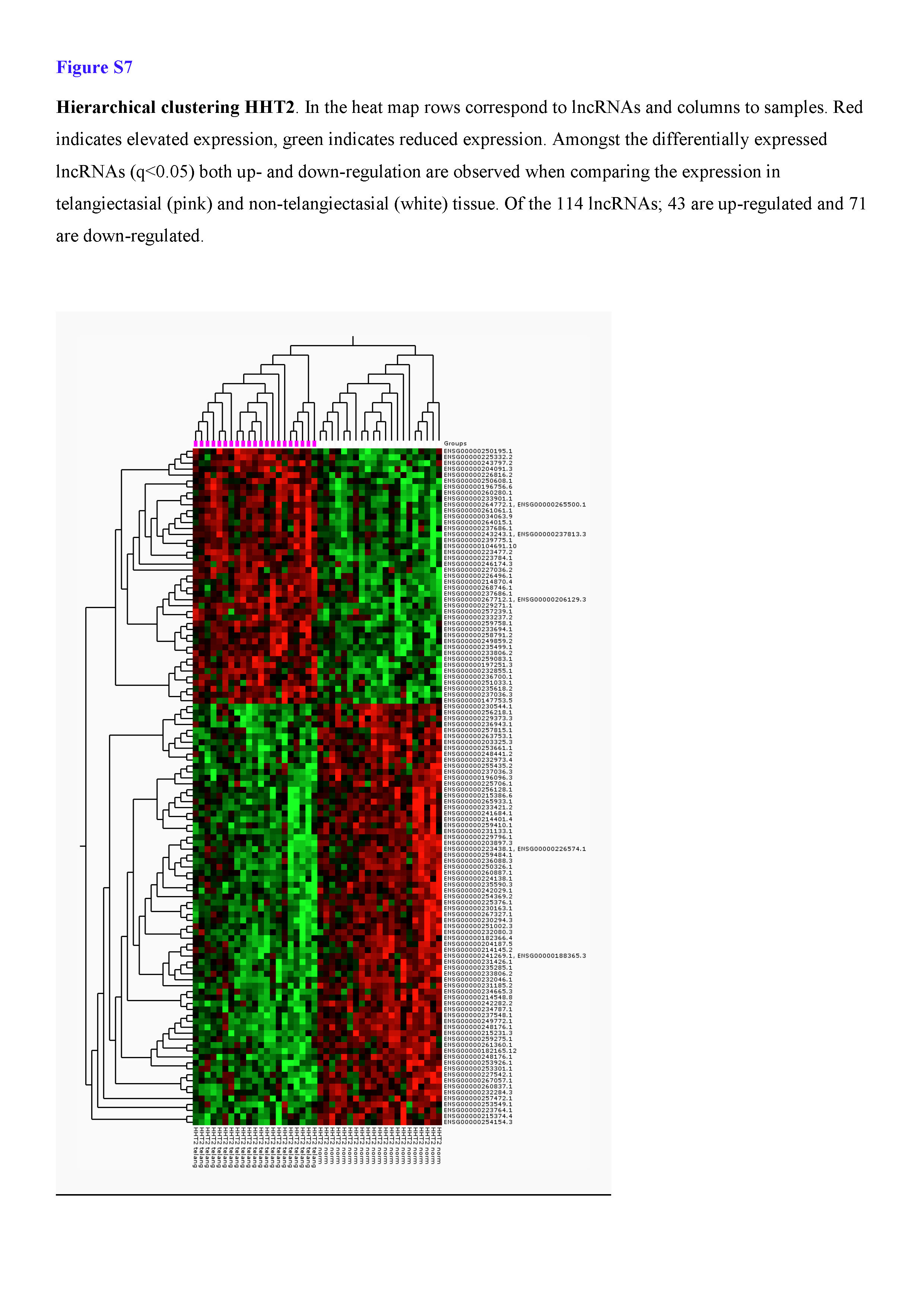

Supplement: Figure S7 — Hierarchical clustering HHT2. (TIFF) [file pone.0090272.s007.tif]
