# Supplementary material for: Long Non-Coding RNA Expression Profiles in Hereditary Haemorrhagic Telangiectasia
Source: PLoS One. 2014 Mar 6;9(3):e90272. doi: 10.1371/journal.pone.0090272 (PMC3946172; doi:10.1371/journal.pone.0090272)
Supplement: File S1 — Contains the Tables S4–S9. Table S4. Gene-genomic regions association table for GO term ‘blood vessel morphogenesis’ (HHT1); Table S5. Gene-genomic regions association table for the GO term ‘blood vessel development’ (HHT1); Table S6. Gene-genomic regions association table for GO term ‘vasculogenesis’ (HHT1); Table S7. Gene-genomic regions association table for GO term ‘vasculogenesis’ (HHT2); Table S8. Gene-genomic regions association table for GO term ‘vasculogenesis’ (HHT); and Table S9. Gene-genomic regions association table for GO term ‘blood vessel morphogenesis’ (HHT). (DOCX) [file pone.0090272.s011.docx]

**Tables S4-S9 list the lncRNAs and corresponding genes involved in the GO terms, plus the q-values, foldchanges and the relative positions of the lncRNAs.**

**Tables S4. Gene-genomic regions association table for GO term ‘blood vessel morphogenesis’ (HHT1).**

| **Gene** | **Chromosome** | **Region/ENSG (distance to TSS)** | **q-value (ENSG)** | **Foldchange (ENSG)** |
| --- | --- | --- | --- | --- |
| ***AMOT*** | X | ENSG00000241743.1 (-794142) | 0.147 | **1.09** |
| ***APOB*** | 2 | ENSG00000233005.1 (-551994) | 0.121 | **1.07** |
| ***BMP4*** | 14 | ENSG00000237356.1 (+526396) | 0.101 | **0.92** |
| ***CAV1*** | 7 | ENSG00000243220.1, ENSG00000231210.2 (+64422),  ENSG00000243220.1, ENSG00000231210.2 (+64422),  ENSG00000243220.1, ENSG00000231210.2 (+64422) | 0.031  0.080  0.086 | **1.18**  **1.10**  **1.12** |
| ***CCM2*** | 7 | ENSG00000232956.3 (-15196) | 0.058 | **1.25** |
| ***CXCL12*** | 10 | ENSG00000233395.1 (+443362),  ENSG00000204187.5 (+537133) | 0.063  0.078 | **0.92**  **0.89** |
| ***CXCR4*** | 2 | ENSG00000231890.2 (+114408) | 0.132 | **1.10** |
| ***CYP1B1*** | 2 | ENSG00000232973.4 (-52571) | 0.111 | **0.89** |
| ***FGF1*** | 5 | ENSG00000231185.2 (+187441),  ENSG00000231185.2 (+187441) | 0.071  0.095 | **1.16**  **0.94** |
| ***FN1*** | 2 | ENSG00000235770.1 (-291575),  ENSG00000235770.1 (-291575),  ENSG00000237525.2 (-187446),  ENSG00000237525.2 (-187446),  ENSG00000237525.2 (-187446) | 0.092  0.121  0.058  0.104  0.136 | **1.07**  **1.06**  **1.10**  **0.93**  **0.88** |
| ***FOXF1*** | 16 | ENSG00000268532.1 (-220619),  ENSG00000268388.1 (-18713) | 0.095  0.132 | **1.08**  **1.08** |
| ***FZD4*** | 11 | ENSG00000255471.1 (+46772),  ENSG00000255471.1 (+46772) | 0.021  0.036 | **0.81**  **0.65** |
| ***GREM1*** | 15 | ENSG00000259721.1 (+135) | 0.096 | **1.09** |
| ***HDAC7*** | 12 | ENSG00000257488.1 (-12396) | 0.097 | **0.91** |
| ***HES1*** | 3 | ENSG00000214146.2 (-155626),  ENSG00000214145.2 (+168492) | 0.138  0.097 | **1.14**  **0.91** |
| ***ITGA5*** | 12 | ENSG00000258137.1 (-6409),  ENSG00000258137.1 (-6409) | 0.097  0.144 | **1.20**  **1.19** |
| ***JAG1*** | 20 | ENSG00000230990.1 (-593802) | 0.072 | **1.27** |
| ***JAM3*** | 11 | ENSG00000204241.3 (-29364) | 0.149 | **0.95** |
| ***LEF1*** | 4 | ENSG00000232021.2 (-43225),  ENSG00000232021.2 (-43225) | 0.048  0.103 | **0.88**  **0.87** |
| ***MEIS1*** | 2 | ENSG00000235725.1 (-820526),  ENSG00000232688.1 (+486584),  ENSG00000236780.1 (+733938),  ENSG00000236780.1 (+733938),  ENSG00000236780.1 (+733938),  ENSG00000236780.1 (+733938),  ENSG00000235885.3 (+755861) | 0.057  0.132  0.051  0.051  0.071  0.097  0.144 | **1.14**  **1.11**  **0.59**  **0.57**  **0.60**  **0.63**  **1.09** |
| ***NOX5*** | 15 | ENSG00000212766.5, ENSG00000259222.1 (+71737) | 0.125 | **0.93** |
| ***NRXN1*** | 2 | ENSG00000231918.1 (-687723) | 0.148 | **0.95** |
| ***PRKX*** | X | ENSG00000236513.1 (-916752) | 0.083 | **1.08** |
| ***PROK2*** | 3 | ENSG00000243083.1 (-377589),  ENSG00000241163.2 (-353727) | 0.087  0.141 | **1.09**  **1.20** |
| ***PRSS23*** | 11 | ENSG00000255471.1 (+108177),  ENSG00000255471.1 (+108177) | 0.021  0.036 | **0.81**  **0.65** |
| ***RASA1*** | 5 | ENSG00000249061.1 (-215736),  ENSG00000249061.1 (-215736),  ENSG00000249061.1 (-215736) | 0.071  0.103  0.129 | **1.16**  **0.92**  **1.12** |
| ***SOX4*** | 6 | ENSG00000260455.1 (+547332),  ENSG00000260455.1 (+547332),  ENSG00000260455.1 (+547332),  ENSG00000260455.1 (+547332) | 0.073  0.092  0.092  0.129 | **1.11**  **1.09**  **1.12**  **1.08** |
| ***STAB2*** | 12 | ENSG00000257703.1 (-457478) | 0.031 | **0.92** |
| ***T*** | 6 | ENSG00000256956.2 (+403575) | 0.072 | **1.16** |
| ***TGFB2*** | 1 | ENSG00000225561.1 (+569137) | 0.133 | **1.13** |
| ***THBS1*** | 15 | ENSG00000259345.1 (-434820),  ENSG00000261136.1 (+203015) | 0.144  0.144 | **0.93**  **1.17** |
| ***TIPARP*** | 3 | ENSG00000240875.1 (+105540)  ENSG00000241544.1 (+408530)  ENSG00000243629.1 (+425672) | 0.056  0.150  0.031 | **1.08**  **1.15**  **0.87** |
| ***WARS*** | 14 | ENSG00000258620.1 (-30763) | 0.082 | **0.92** |
| ***WNT7A*** | 3 | ENSG00000224514.1 (+181454) | 0.074 | **1.10** |
| ***ZC3H12A*** | 1 | ENSG00000233621.1 (-9873) | 0.092 | **0.92** |
| ***ZFPM2*** | 8 | ENSG00000251003.2 (+601466) | 0.045 | **1.23** |
| ***ZMIZ1*** | 10 | ENSG00000230417.5 (-607181) | 0.147 | **1.06** |

**Table S5. Gene-genomic regions association table for the GO term ‘blood vessel development’ (HHT1).**

| **Gene** | **Chromosome** | **Region/ENSG (distance to TSS)** | **q-value (ENSG)** | **Foldchange (ENSG)** |
| --- | --- | --- | --- | --- |
| ***AMOT*** | X | ENSG00000241743.1 (-794142) | 0.147 | **1.095** |
| ***APOB*** | 2 | ENSG00000233005.1 (-551994) | 0.121 | **1.07** |
| ***BMP4*** | 14 | ENSG00000237356.1 (+526396) | 0.101 | **0.92** |
| ***CAV1*** | 7 | ENSG00000243220.1, NSG00000231210.2 (+64422),  ENSG00000243220.1, NSG00000231210.2 (+64422),  ENSG00000243220.1, NSG00000231210.2 (+64422) | 0.031  0.080  0.086 | **1.18**  **1.10**  **1.12** |
| ***CCM2*** | 7 | ENSG00000232956.3 (-15196) | 0.058 | **1.25** |
| ***CDH5*** | 16 | ENSG00000261742.1 (-936222) | 0.071 | **0.89** |
| ***CXCL12*** | 10 | ENSG00000233395.1 (+443362),  ENSG00000204187.5 (+537133) | 0.063  0.078 | **0.92**  **0.89** |
| ***CXCR4*** | 2 | ENSG00000231890.2 (+114408) | 0.132 | **1.10** |
| ***CYP1B1*** | 2 | ENSG00000232973.4 (-52571) | 0.111 | **0.89** |
| ***FGF1*** | 5 | ENSG00000231185.2 (+187441),  ENSG00000231185.2 (+187441) | 0.071  0.095 | **1.16**  **0.94** |
| ***FN1*** | 2 | ENSG00000235770.1 (-291575),  ENSG00000235770.1 (-291575),  ENSG00000237525.2 (-187446),  ENSG00000237525.2 (-187446),  ENSG00000237525.2 (-187446) | 0.092  0.121  0.058  0.104  0.136 | **1.07**  **1.06**  **1.10**  **0.93**  **0.88** |
| ***FOXF1*** | 16 | ENSG00000268532.1 (-220619),  ENSG00000268388.1 (-18713) | 0.095  0.132 | **1.08**  **1.08** |
| ***FOXO1*** | 13 | ENSG00000215483.4 (+200597) | 0.074 | **1.11** |
| ***FZD4*** | 11 | ENSG00000255471.1 (+46772),  ENSG00000255471.1 (+46772) | 0.021  0.036 | **0.81**  **0.65** |
| ***GREM1*** | 15 | ENSG00000259721.1 (+135) | 0.096 | **1.09** |
| ***HDAC7*** | 12 | ENSG00000257488.1 (-12396) | 0.097 | **0.91** |
| ***HES1*** | 3 | ENSG00000214146.2 (-155626),  ENSG00000214145.2 (+168492) | 0.138  0.097 | **1.14**  **0.91** |
| ***ITGA5*** | 12 | ENSG00000258137.1 (-6409),  ENSG00000258137.1 (-6409) | 0.097  0.144 | **1.20**  **1.19** |
| ***JAG1*** | 20 | ENSG00000230990.1 (-593802) | 0.072 | **1.27** |
| ***JAM3*** | 11 | ENSG00000204241.3 (-29364) | 0.149 | **0.95** |
| ***LEF1*** | 4 | ENSG00000232021.2 (-43225),  ENSG00000232021.2 (-43225) | 0.048  0.103 | **0.88**  **0.87** |
| ***MEF2C*** | 5 | ENSG00000248309.1 (-270758),  ENSG00000245526.3 (+304811) | 0.122  0.072 | **1.09**  **1.11** |
| ***MEIS1*** | 2 | ENSG00000235725.1 (-820526),  ENSG00000232688.1 (+486584),  ENSG00000236780.1 (+733938),  ENSG00000236780.1 (+733938),  ENSG00000236780.1 (+733938),  ENSG00000236780.1 (+733938),  ENSG00000235885.3 (+755861) | 0.057  0.132  0.051  0.051  0.071  0.097  0.144 | **1.14**  **1.11**  **0.59**  **0.57**  **0.60**  **0.63**  **1.09** |
| ***NOX5*** | 15 | ENSG00000212766.5, ENSG00000259222.1  (+71737) | 0.125 | **0.93** |
| ***NRXN1*** | 2 | ENSG00000231918.1 (-687723) | 0.148 | **0.95** |
| ***PRKX*** | X | ENSG00000236513.1 (-916752) | 0.083 | **1.08** |
| ***PROK2*** | 3 | ENSG00000243083.1 (-377589),  ENSG00000241163.2 (-353727) | 0.087  0.141 | **1.09**  **1.20** |
| ***PRSS23*** | 11 | ENSG00000255471.1 (+108177),  ENSG00000255471.1 (+108177) | 0.021  0.036 | **0.81**  **0.65** |
| ***RASA1*** | 5 | ENSG00000249061.1 (-215736),  ENSG00000249061.1 (-215736),  ENSG00000249061.1 (-215736) | 0.071  0.103  0.129 | **1.16**  **0.92**  **1.12** |
| ***SOX4*** | 6 | ENSG00000260455.1 (+547332),  ENSG00000260455.1 (+547332),  ENSG00000260455.1 (+547332),  ENSG00000260455.1 (+547332) | 0.073  0.092  0.092  0.129 | **1.11**  **1.09**  **1.12**  **1.08** |
| ***STAB2*** | 12 | ENSG00000257703.1 (-457478) | 0.031 | **0.92** |
| ***T*** | 6 | ENSG00000256956.2 (+403575) | 0.072 | **1.16** |
| ***TGFB2*** | 1 | ENSG00000225561.1 (+569137) | 0.133 | **1.13** |
| ***THBS1*** | 15 | ENSG00000259345.1 (-434820),  ENSG00000261136.1 (+203015) | 0.144  0.144 | **0.93**  **1.17** |
| ***TIPARP*** | 3 | ENSG00000240875.1 (+105540)  ENSG00000241544.1 (+408530)  ENSG00000243629.1 (+425672) | 0.056  0.150  0.031 | **1.08**  **1.15**  **0.87** |
| ***WARS*** | 14 | ENSG00000258620.1 (-30763) | 0.082 | **0.92** |
| ***WNT2*** | 7 | ENSG00000226367.1 (+214013) | 0.130 | **0.93** |
| ***WNT7A*** | 3 | ENSG00000224514.1 (+181454) | 0.074 | **1.10** |
| ***ZC3H12A*** | 1 | ENSG00000233621.1 (-9873) | 0.092 | **0.92** |
| ***ZFPM2*** | 8 | ENSG00000251003.2 (+601466) | 0.045 | **1.23** |
| ***ZMIZ1*** | 10 | ENSG00000230417.5 (-607181) | 0.147 | **1.06** |

**Table S6. Gene-genomic regions association table for GO term ‘vasculogenesis’ (HHT1).**

| **Gene** | **Chromosome** | **Region/ENSG (distance to TSS)** | **q-value (ENSG)** | **Foldchange (ENSG)** |
| --- | --- | --- | --- | --- |
| ***AMOT*** | X | ENSG00000241743.1 (-794142) | 0.147 | **1.09** |
| ***CAV1*** | 7 | ENSG00000243220.1,ENSG00000231210.2 (+64422),  ENSG00000243220.1, ENSG00000231210.2 (+64422),  ENSG00000243220.1, ENSG00000231210.2 (+64422) | 0.031  0.080  0.086 | **1.18**  **1.10**  **1.12** |
| ***CCM2*** | 7 | ENSG00000232956.3 (-15196) | 0.058 | **1.25** |
| ***FOXF1*** | 16 | ENSG00000268532.1 (-220619),  ENSG00000268388.1 (-18713) | 0.095  0.132 | **1.08**  **1.08** |
| ***FZD4*** | 11 | ENSG00000255471.1 (+46772),  ENSG00000255471.1 (+46772) | 0.021  0.036 | **0.81**  **0.65** |
| ***HDAC7*** | 12 | ENSG00000257488.1 (-12396) | 0.097 | **0.91** |
| ***PRSS23*** | 11 | ENSG00000255471.1 (+108177),  ENSG00000255471.1 (+108177) | 0.021  0.036 | **0.81**  **0.65** |
| ***RASA1*** | 5 | ENSG00000249061.1 (-215736),  ENSG00000249061.1 (-215736),  ENSG00000249061.1 (-215736) | 0.071  0.103  0.129 | **1.16**  **0.92**  **1.12** |
| ***T*** | 6 | ENSG00000256956.2 (+403575) | 0.072 | **1.16** |
| ***TIPARP*** | 3 | ENSG00000240875.1 (+105540)  ENSG00000241544.1 (+408530)  ENSG00000243629.1 (+425672) | 0.056  0.150  0.031 | **1.08**  **1.15**  **0.87** |
| ***WNT7A*** | 3 | ENSG00000224514.1 (+181454) | 0.074 | **1.99** |
| ***ZFPM2*** | 8 | ENSG00000251003.2 (+601466) | 0.045 | **1.23** |
| ***ZMIZ1*** | 10 | ENSG00000230417.5 (-607181) | 0.147 | **1.06** |

**Table S7. Gene-genomic regions association table for GO term ‘vasculogenesis’ (HHT2).**

| **Gene** | **Chromosome** | **Region/ENSG (distance to TSS)** | **q-value (ENSG)** | **Foldchange (ENSG)** |
| --- | --- | --- | --- | --- |
| ***CAV1*** | 7 | ENSG00000243220.1,ENSG00000231210.2 (+64422) | 0.141 | **1.08** |
| ***CCM2*** | 7 | ENSG00000232956.3 (-15196),  ENSG00000232956.3 (-15196) | 0.055  0.080 | **1.16**  **1.23** |
| ***CITED2*** | 6 | ENSG00000231426.1 (-483288) | 0.025 | **0.92** |
| ***FOXF1*** | 16 | ENSG00000268388.1 (-18713) | 0.065 | **1.09** |
| ***FZD4*** | 11 | ENSG00000255471.1 (+46772),  ENSG00000255471.1 (+46772),  ENSG00000255471.1 (+46772) | 0.077  0.110  0.129 | **0.81**  **0.91**  **0.84** |
| ***PRSS23*** | 11 | ENSG00000255471.1 (+108177),  ENSG00000255471.1 (+108177),  ENSG00000255471.1 (+108177) | 0.077  0.110  0.129 | **0.81**  **0.91**  **0.84** |
| ***RASA1*** | 5 | ENSG00000249061.1 (-215736)  ENSG00000249061.1 (-215736) | 0.082  0.148 | **1.08**  **0.86** |
| ***SHH*** | 7 | ENSG00000225666.1 (-860485),  ENSG00000241558.1, ENSG00000182648.7 (-744121) | 0.088  0.129 | **1.09**  **0.88** |
| ***T*** | 6 | ENSG00000256956.2 (+402852) | 0.100 | **1.28** |
| ***TIPARP*** | 3 | ENSG00000240875.1 (+105540),  ENSG00000243629.1 (+425672),  ENSG00000243629.1 (+425672) | 0.111  0.134  0.142 | **0.86**  **1.05**  **0.84** |
| ***VEGFA*** | 6 | ENSG00000237686.1 (+264979),  ENSG00000237686.1 (+264979) | 0.046  0.050 | **1.12**  **1.24** |
| ***WNT7B*** | 22 | ENSG00000231010.1 (-79822) | 0.127 | **0.90** |
| ***ZFPM2*** | 8 | ENSG00000254615.2 (+952603),  ENSG00000254615.2 (+952603) | 0.121  0.138 | **1.14**  **1.21** |
| ***ZMIZ1*** | 10 | ENSG00000230417.5 (-607181),  ENSG00000230417.5 (-607181) | 0.052  0.725 | **0.91**  **1.11** |

**Table S8. Gene-genomic regions association table for GO term ‘vasculogenesis’ (HHT).**

| **Gene** | **Chromosome** | **Region/ENSG (distance to TSS)** | **q-value (ENSG)** | **Foldchange (ENSG)** |
| --- | --- | --- | --- | --- |
| ***CAV1*** | 7 | ENSG00000243220.1,ENSG00000231210.2 (+64422),  ENSG00000243220.1,ENSG00000231210.2 (+64422)  ENSG00000243220.1,ENSG00000231210.2 (+64422) | 0.006  0.007  0.049 | **1.00**  **1.12**  **1.07** |
| ***CCM2*** | 7 | ENSG00000232956.3 (-15196),  ENSG00000232956.3 (-15196) | 0.001  0.016 | **1.20**  **1.16** |
| ***CITED2*** | 6 | ENSG00000231426.1 (-483288) | 0.009 | **0.93** |
| ***FOXF1*** | 16 | ENSG00000268532.1 (-220619),  ENSG00000268388.1 (-18713) | 0.023  0.023 | **1.06**  **1.09** |
| ***FZD4*** | 11 | ENSG00000255471.1 (+46772),  ENSG00000255471.1 (+46772)  ENSG00000255471.1 (+46772) | 0.0004  0.003  0.038 | **0.81**  **0.68**  **0.93** |
| ***HDAC7*** | 12 | ENSG00000257488.1 (-12396) | 0.008 | **0.92** |
| ***PRSS23*** | 11 | ENSG00000255471.1 (+108177),  ENSG00000255471.1 (+108177)  ENSG00000255471.1 (+108177) | 0.0004  0.003  0.038 | **0.81**  **0.68**  **0.93** |
| ***RASA1*** | 5 | ENSG00000249061.1 (-215736) | 0.007 | **1.10** |
| ***SHH*** | 7 | ENSG00000225666.1 (-860485) | 0.036 | **1.04** |
| ***TIPARP*** | 3 | ENSG00000240875.1 (+105540),  ENSG00000240875.1 (+105540),  ENSG00000243629.1 (+425672) | 0.019  0.021  0.007 | **0.89**  **1.06**  **0.91** |
| ***VEGFA*** | 6 | ENSG00000237686.1 (+264979)  ENSG00000237686.1 (+264979) | 0.012  0.049 | **1.16**  **1.07** |
| ***WNT7A*** | 3 | ENSG00000224514.1 (+181454) | 0.039 | **1.06** |
| ***ZFPM2*** | 8 | ENSG00000251003.2 (+601466) | 0.037 | **1.13** |
| ***ZMIZ1*** | 10 | ENSG00000230417.5 (-607181) | 0.006 | **1.09** |

**Table S9. Gene-genomic regions association table for GO term ‘blood vessel morphogenesis’ (HHT).**

| **Gene** | **Chromosome** | **Region/ENSG (distance to TSS)** | **q-value (ENSG)** | **Foldchange (ENSG)** |
| --- | --- | --- | --- | --- |
| ***ANGPT2*** | 8 | ENSG00000249898.3 (-100079) | 0.0199 | **1.13** |
| ***APOB*** | 2 | ENSG00000233005.1 (-551994) | 0.029 | **1.06** |
| ***BMP4*** | 14 | ENSG00000237356.1 (+526396) | 0.005 | **0.93** |
| ***CAV1*** | 7 | ENSG00000243220.1,ENSG00000231210.2 (+64422)  ENSG00000243220.1,ENSG00000231210.2 (+64422)  ENSG00000243220.1,ENSG00000231210.2 (+64422) | 0.006  0.007  0.049 | **1.01**  **1.12**  **1.07** |
| ***CCM2*** | 7 | ENSG00000232956.3 (-15196),  ENSG00000232956.3 (-15196) | 0.001  0.016 | **1.20**  **1.16** |
| ***CITED2*** | 6 | ENSG00000231426.1 (-483288) | 0.009 | **0.93** |
| ***CXCL12*** | 10 | ENSG00000233395.1 (+443362),  ENSG00000204187.5 (+537133) | 0.002  0.001 | **0.92**  **0.91** |
| ***CYP1B1*** | 2 | ENSG00000232973.4 (-52571) | 0.002 | **0.90** |
| ***FGF1*** | 5 | ENSG00000231185.2 (+187441),  ENSG00000231185.2 (+187441) | 0.001  0.026 | **0.93**  **0.93** |
| ***FN1*** | 2 | ENSG00000235770.1 (-291575),  ENSG00000235770.1 (-291575),  ENSG00000237525.2 (-187446),  ENSG00000237525.2 (-187446),  ENSG00000230838.1 (-282666) | 0.010  0.038  0.003  0.007  0.048 | **0.93**  **1.05**  **1.08**  **0.94**  **1.22** |
| ***FOXF1*** | 16 | ENSG00000268532.1 (-220619),  ENSG00000268388.1 (-18713) | 0.023  0.023 | **1.06**  **1.09** |
| ***FZD4*** | 11 | ENSG00000255471.1 (+46772),  ENSG00000255471.1 (+46772)  ENSG00000255471.1 (+46772) | 0.0004  0.003  0.048 | **0.81**  **0.68**  **1.22** |
| ***HDAC7*** | 12 | ENSG00000257488.1 (-12396) | 0.008 | **0.92** |
| ***HES1*** | 3 | ENSG00000214145.2 (+168492) | 0.004 | **0.94** |
| ***ITGB1*** | 10 | ENSG00000229656.2 (-62109) | 0.046 | **1.09** |
| ***LEF1*** | 4 | ENSG00000232021.2 (-43225),  ENSG00000232021.2 (-43225) | 0.001  0.003 | **0.90**  **0.87** |
| ***MEIS1*** | 2 | ENSG00000204929.6 (-767275),  ENSG00000232046.1 (+216694),  ENSG00000236780.1 (+733938),  ENSG00000236780.1 (+733938),  ENSG00000236780.1 (+733938),  ENSG00000235885.3 (+755861) | 0.041  0.001  0.029  0.032  0.034  0.009 | **1.05**  **0.92**  **0.66**  **0.66**  **0.68**  **1.10** |
| ***NR2F2*** | 15 | ENSG00000259275.1 (+48104) | 0.003 | **0.92** |
| ***NRP1*** | 10 | ENSG00000229656.2 (+314431) | 0.046 | **1.0** |
| ***NRXN1*** | 2 | ENSG00000231918.1 (-687723) | 0.010 | **0.94** |
| ***PROK2*** | 3 | ENSG00000243083.1 (-377589) | 0.004 | **1.08** |
| ***PRSS23*** | 11 | ENSG00000255471.1 (+108177),  ENSG00000255471.1 (+108177)  ENSG00000255471.1 (+108177) | 0.0004  0.003  0.038 | **0.81**  **0.68**  **0.93** |
| ***RASA1*** | 5 | ENSG00000249061.1 (-215736) | 0.007 | **1.10** |
| ***SHH*** | 7 | ENSG00000225666.1 (-860485) | 0.036 | **1.04** |
| ***SOX4*** | 6 | ENSG00000260455.1 (+547332),  ENSG00000260455.1 (+547332),  ENSG00000260455.1 (+547332) | 0.025  0.029  0.040 | **0.94**  **1.06**  **0.92** |
| ***STAB2*** | 12 | ENSG00000257703.1 (-457478) | 0.010 | **0.94** |
| ***TGFB2*** | 1 | ENSG00000225561.1 (+569137) | 0.032 | **1.08** |
| ***THBS1*** | 15 | ENSG00000259345.1 (-434820),  ENSG00000259345.1 (-434820) | 0.009  0.010 | **1.09**  **0.93** |
| ***TIPARP*** | 3 | ENSG00000240875.1 (+105540),  ENSG00000240875.1 (+105540),  ENSG00000243629.1 (+425672) | 0.019  0.021  0.007 | **0.89**  **1.06**  **0.91** |
| ***VEGFA*** | 6 | ENSG00000237686.1 (+264979)  ENSG00000237686.1 (+264979) | 0.012  0.049 | **1.16**  **1.07** |
| ***WNT7A*** | 12 | ENSG00000224514.1 (+181454) | 0.042 | **0.91** |
| ***ZFPM2*** | 8 | ENSG00000251003.2 (+601466) | 0.037 | **1.12** |
| ***ZMIZ1*** | 10 | ENSG00000230417.5 (-607181) | 0.006 | **1.09** |
